# Single‐nucleus and spatial transcriptome reveal adrenal homeostasis in normal and tumoural adrenal glands

**DOI:** 10.1002/ctm2.1798

**Published:** 2024-08-21

**Authors:** Barbara Altieri, A. Kerim Secener, Somesh Sai, Cornelius Fischer, Silviu Sbiera, Panagiota Arampatzi, Stefan Kircher, Sabine Herterich, Laura‐Sophie Landwehr, Sarah N. Vitcetz, Caroline Braeuning, Martin Fassnacht, Cristina L. Ronchi, Sascha Sauer

**Affiliations:** ^1^ Division of Endocrinology and Diabetes Department of Internal Medicine I University Hospital University of Würzburg Würzburg Germany; ^2^ Max Delbrück Center for Molecular Medicine Berlin Germany; ^3^ Berlin Institute of Health Berlin Germany; ^4^ Department of Biology Chemistry and Pharmacy Institute of Biochemistry Free University Berlin Berlin Germany; ^5^ Core Unit SysMed University of Würzburg Würzburg Germany; ^6^ Institute of Pathology University of Würzburg Würzburg Germany; ^7^ Central Laboratory University Hospital Würzburg Würzburg Germany; ^8^ Institute of Metabolism and System Research University of Birmingham Edgabston, Birmingham UK

**Keywords:** adenoma, adrenal homeostasis, adrenocortical tumour, cortisol secretion, CTNNB1, heterogeneity, microenvironment, spatial transcriptome, tumorigenesis

## Abstract

The human adrenal gland is a complex endocrine tissue. Studies on adrenal renewal have been limited to animal models or human foetuses. Enhancing our understanding of adult human adrenal homeostasis is crucial for gaining insights into the pathogenesis of adrenal diseases, such as adrenocortical tumours.

Here, we present a comprehensive cellular genomics analysis of the adult human normal adrenal gland, combining single‐nuclei RNA sequencing and spatial transcriptome data to reconstruct adrenal gland homeostasis. As expected, we identified primary cells of the various zones of the adrenal cortex and medulla, but we also uncovered additional cell types. They constitute the adrenal microenvironment, including immune cells, mostly composed of a large population of M2 macrophages, and new cell populations, including different subpopulations of vascular‐endothelial cells and cortical‐neuroendocrine cells. Utilizing spatial transcriptome and pseudotime trajectory analysis, we support evidence of the centripetal dynamics of adrenocortical cell maintenance and the essential role played by Wnt/β‐catenin, sonic hedgehog, and fibroblast growth factor pathways in the adult adrenocortical homeostasis. Furthermore, we compared single‐nuclei transcriptional profiles obtained from six healthy adrenal glands and twelve adrenocortical adenomas. This analysis unveiled a notable heterogeneity in cell populations within the adenoma samples. In addition, we identified six distinct adenoma‐specific clusters, each with varying distributions based on steroid profiles and tumour mutational status.

Overall, our results provide novel insights into adrenal homeostasis and molecular mechanisms potentially underlying early adrenocortical tumorigenesis and/or autonomous steroid secretion. Our cell atlas represents a powerful resource to investigate other adrenal‐related pathologies.

## Graphical abstract

- This study unveils the cellular diversity within the adult human adrenal gland using single‐nuclei RNA sequencing and spatial transcriptome data.
- Analysis of the adult human adrenal gland identifies new cell populations and key pathways governing adrenal homeostasis.
- Significant cellular and transcriptomic heterogeneity is discovered when comparing adrenocortical adenomas to healthy adrenal glands, shedding light on adrenocortical tumorigenesis and steroid hormones regulation.

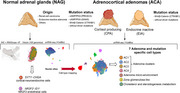

## 1 INTRODUCTION

The human adrenal gland is a complex endocrine tissue that maintains homeostasis by responding to various physiological stimuli by secreting steroid hormones and catecholamines. The adult adrenal gland consists of two functionally distinct parts, the cortex and the medulla, which develop from different embryological origins.^1^ The adrenal cortex is organized in three zones bearing diverse morphological and functional characteristics, for example, zona glomerulosa (ZG), fasciculata (ZF) and reticularis (ZR) involved in mineralocorticoid, glucocorticoid, and androgen synthesis and secretion, respectively. The centre of the adrenal gland is occupied by catecholamine‐secreting medulla, originating from neural crest cells.

Several studies conducted using transgenic mouse and rat models have elucidated aspects of adrenal development and homeostasis.^2^ According to these studies, the adrenocortical zonation follows a centripetal differentiation and is driven by several signalling pathways, including the Wnt/β‐catenin, the sonic hedgehog (SHH), the cAMP/protein kinase A (PKA), the insulin‐like growth factor (IGF), and the fibroblast growth factor (FGF) pathways.^3^, ^4^, ^5^, ^6^, ^7^ The dysregulation of these signalling pathways is likely involved in human adrenal tumorigenesis.^8^ However, since most of these studies have been performed on mice models, their findings may not be directly applicable to humans.

The incidence of adrenal tumours increases with age. The most common type is benign adrenocortical adenomas (ACAs), while malignant adrenocortical carcinomas (ACCs) are rare.^9^ Benign tumours are often incidentally detected and are predominantly endocrine‐inactive adenomas (EIA).^10^, ^11^ However, some can lead to mild or overt autonomous cortisol secretion (cortisol‐producing adenomas, CPA). In recent years, comprehensive genomics studies have identified alterations in different signalling pathways involved in adrenocortical tumorigenesis and autonomous cortisol secretion, including Wnt/β‐catenin‐, Rb/p53‐, IGF‐ and cAMP/PKA‐signalling pathways.^10^, ^11^, ^12^, ^13^, ^14^, ^15^, ^16^ However, the molecular mechanisms underlying the pathogenesis of a vast percentage of these tumours remain unexplained. Therefore, a deeper understanding of normal adrenal homeostasis and self‐maintenance is urgently required.

The use of cell atlases created from single‐cell/single‐nuclei RNA‐sequencing (sc/snRNA‐seq), along with spatial transcriptomics, has significantly improved our understanding of the cellular heterogeneity found in both normal and tumoral tissues. This enhanced knowledge has deepened our insights into the process of tumorigenesis.^17^, ^18^, ^19^ Recently, multiple scRNA‐seq studies have concentrated on understanding the developmental stages of both mouse^20^, ^21^, ^22^, ^23^ and human adrenal glands.^24^, ^25^ These studies have highlighted the presence of rare cell types, such as multipotent Schwann cell precursors^20^, ^22^, ^26^, ^27^ and postnatal progenitor populations of chromaffin cells.^20^ However, these studies mostly focused on the embryonic^28^ or fetal development,^25^ as well as the adrenal gland's response to stress,^23^ without providing a comprehensive characterization of cell types in the adult adrenal cortex. Up to now, only one study has explored the human adult adrenal microenvironment, uncovering immune activation within a subset of endothelial cells.^29^ Very recently, our group investigated the transcriptome heterogeneity of ACC at the snRNA‐seq level.^30^ Nevertheless, a comprehensive characterization of the normal adult adrenal cell types is still largely unexplored.

Therefore, we aimed to contribute the first comprehensive transcriptome analysis of the cortex of the adult human normal adrenal gland (NAG) at single nuclei resolution including spatial information. Furthermore, we integrated our data with snRNA‐seq datasets from a clinical ACA cohort—comprised of EIA and CPA patient samples—to reveal tumor‐specific composition and cell subpopulations.

## 2 RESULTS

### 2.1 Single‐nuclei transcriptome sequencing of NAGs

**FIGURE 1:**
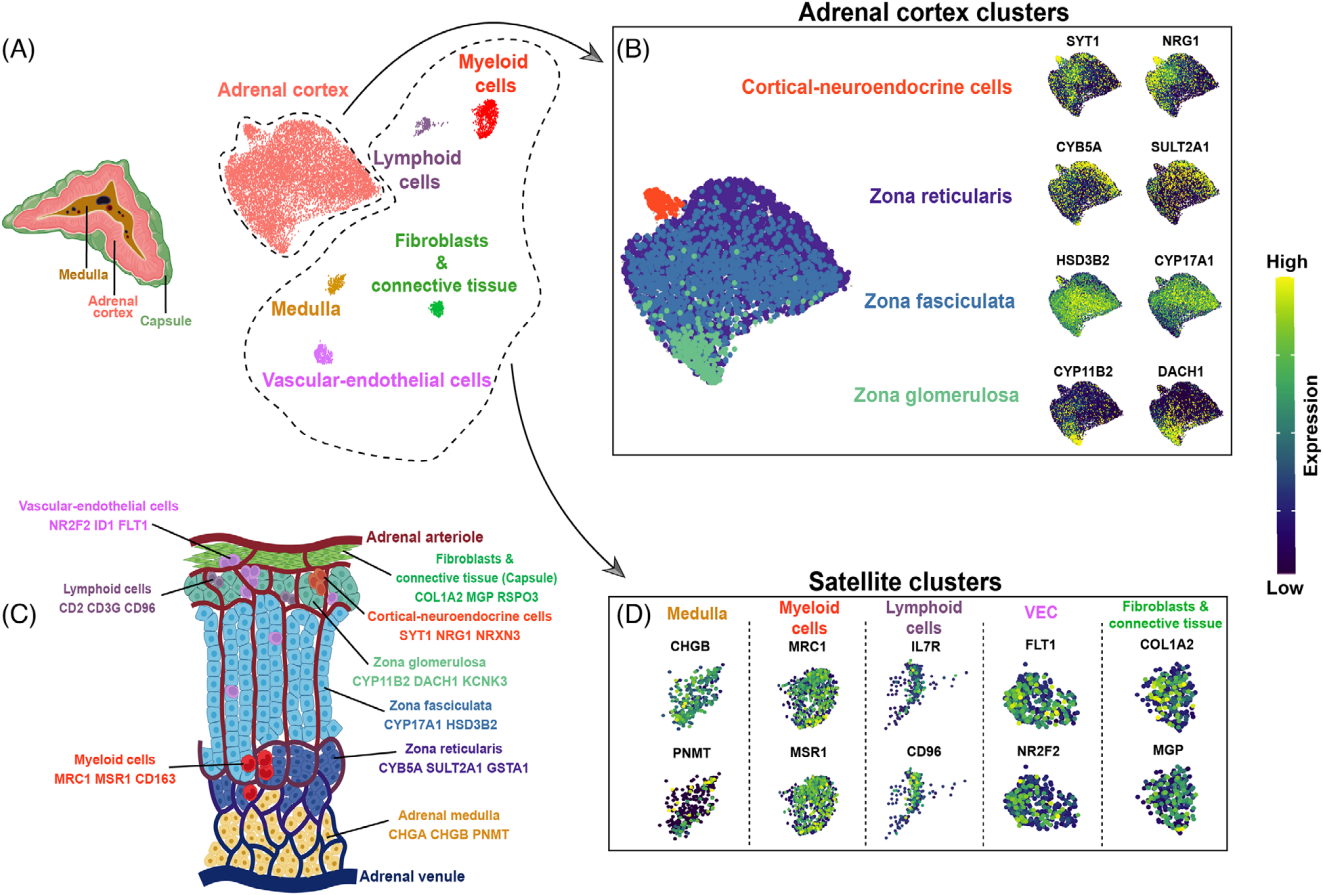
**Single‐nucleus analysis of the adult human normal adrenal gland**. (A) Left: Transverse section depicting the three major adrenal zones (capsule, adrenal cortex, and medulla). Right: UMAP (Uniform Manifold Approximation and Projection) representation of the six integrated single‐nuclei transcriptomic datasets (clusters were annotated by using known marker genes and DEG analysis). (B) Adrenal cortex clusters: left: UMAP representation of the adrenal cortex clusters with complete zonation inferred by module scoring (see Methods); right: feature expression plots representing genes specific for zonae reticularis, fasciculata, glomerulosa and cortical‐neuroendocrine cells (scale represents log normalized expression values). (C) Anatomical sketch of the adult human normal adrenal gland: the displayed markers for each identified cell type are based on DGE analysis in single‐cell transcriptome data, available literature (see text for references) and in‐situ validations by immunohistochemistry. The image was created with BioRender. (D) Satellite clusters. Feature expression plots representing genes used in the annotation of the following clusters: medulla, myeloid cells, lymphoid cells, vascular‐endothelial cells (VEC), and fibroblasts & connective tissue (scale represents log normalized expression values).

We sequenced 11,931 nuclei from six NAGs (Table 1 and Figure S1), obtaining an average depth of 25 million reads per sample. In an unsupervised cluster analysis, we identified six main cell clusters with distinct gene expression signatures. The identity of each cluster was assigned by cross‐referencing upregulated transcripts with canonical markers from the literature. The dominant cluster was populated by classical adrenocortical cell types (Figure 1A), including subclusters of ZG, ZF and ZR, as well as a new subcluster that we termed “cortical‐neuroendocrine cells” (CNC) (Figure 1B). Highly expressed genes typical for these cortex subclusters are shown in Figure 1C. The remaining “satellite” clusters were annotated as medulla, fibroblasts & connective tissue (FC), vascular‐endothelial cells (VEC), myeloid cells (MC) and lymphoid cells (LC) (Figure 1D). The top 100 differentially expressed genes (DEGs) defining the individual clusters of the NAG are listed in Table S1.

**TABLE 1:** Overview of demographic details and transcriptome data of the six adult human normal adrenal glands included in the single nuclei RNA‐sequencing.

ID	Reason for surgery	Sex	Age	Adrenal side	# of QC'ed nuclei	Mean Counts	Mean features per nucleus	Total reads per sample
NAGe‐1	EIA	F	54	Right	2851	555	461	25.668.558
NAGe‐2	EIA	F	49	Right	921	1127	849	22.781.046
NAGe‐3	EIA	M	71	Left	2002	627	489	27.786.791
NAGr‐1	RCC	M	56	Left	2323	760	585	24.765.006
NAGr‐2	RCC	M	81	Left	2073	793	631	25.215.884
NAGr‐3	RCC	M	68	Left	1761	663	504	20.974.508

Abbreviations: EIA, endocrine‐inactive tumour; F, female; M, male; n.a., not available; NAG, normal adrenal gland; RCC, renal cell carcinoma; #, number.

### 2.2 Zonation of the adrenal cortex and adrenal medulla

#### 2.2.1 Adrenocortical cell types

**FIGURE 2:**
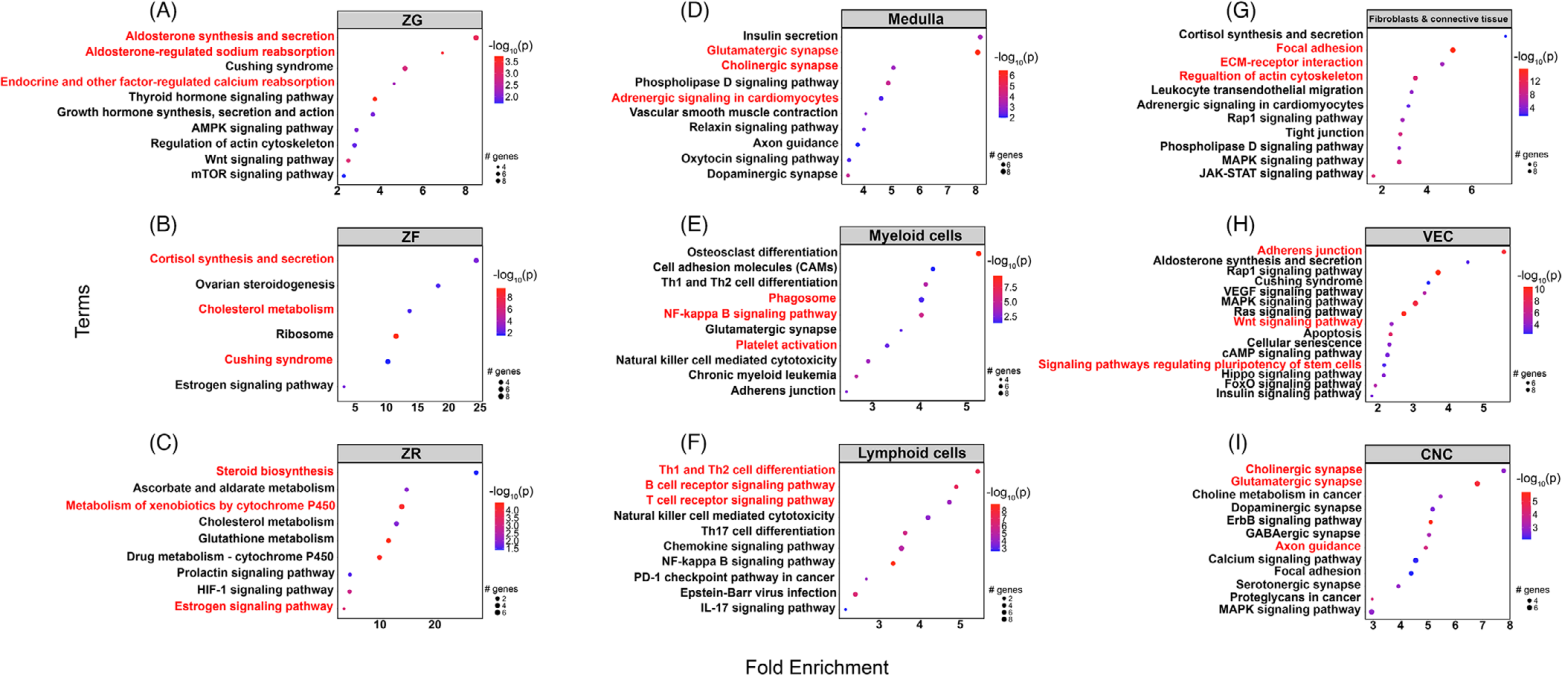
**Gene set enrichment analysis of single‐nuclei transcriptome in adult human normal adrenal glands**. Gene set enrichment analysis was performed using pathfindR (KEGG) and results for each cluster are represented separately. The dot size varies with the quantity of observed significant genes that constitute their representative enriched term. The colour scale represents the –log10(p) value. The x‐axis indicates fold enrichment, while the y‐axis covers the enriched terms within the representative clusters. Abbreviations: CNC, cortical‐neuroendocrine cells; VEC, vascular‐endothelial cells; ZF, zona fasciculata; ZG, zona glomerulosa; ZR, zona reticularis.

Within the adrenal cortex cluster, different cell subpopulations showed high expression of genes encoding for key steroidogenic enzymes representing the three cortex zones (Figure 1B, Figure S2A–C and Table S1). Cells from the ZG subcluster showed high expression of *CYP11B2*, as well as *DACH1* and *ANO4*, all previously described to be highly selective for this zone.^31^, ^32^, ^33^, ^34^ The ZF was characterized by high expression of *CYP17A1*, *CYP11B1* and *HSD3B2*, whereas we observed elevated expression of *CYB5A*, *SULT2A1* and *GSTA1* in the ZR.^35^, ^36^ Gene enrichment analyses with DEGs from the three adrenocortical zones showed a significant overlap with regard to our annotation. Aldosterone synthesis (fold enrichment [FE] > 8), aldosterone‐regulated sodium reabsorption (FE > 6), endocrine and other factor‐regulated calcium reabsorption (FE > 4), and Wnt signalling (FE > 2) were upregulated in the ZG subcluster, while cortisol synthesis and cholesterol metabolism were clearly overrepresented in the ZF (FE > 23 and > 13, respectively, Figure 2A,B). Steroid biosynthesis (FE > 27), metabolism of xenobiotics by cytochrome P450 (FE > 13), and estrogen signalling (FE > 3) were highly represented in the ZR subcluster (Figure 2C).

To further validate the transcriptomic zonation of the adrenal cortex at the protein level, we performed immunohistochemistry (IHC) of selected mRNA‐based markers (represented via the kernel density^37^ in the “Uniform Manifold Approximation and Projection”, UMAP; Figure S3). Consistent with the expression pattern of the corresponding mRNAs in our single nuclei dataset, immuno‐staining of DACH1 was uniform in the subcapsular region, while CYP11B2 appeared in specific niches (Figure S3B). Moreover, CYP17A1 protein expression was detected in the entire cortex region, corresponding to a higher RNA expression density in ZF with lower expression in the adjacent ZG and ZR clusters (Figure S3C). In the snRNA dataset, both *CYB5A* and *SULT2A1* densities were similarly highlighted in the UMAP, predominantly marking the ZR. However, SULT2A1 also showed low expression in the ZF. This result was confirmed also at the protein level by IHC, where SULT2A1 showed a strong staining in the ZR and comparatively lower intensity staining in the ZF. In contrast, CYB5A staining provided a clear distinction between the ZF and ZR (Figure S3D).

#### 2.2.2 Adreno‐medullary cells

In addition to the cortical cells, we characterized medullary cells as well, which showed an expression pattern typical for chromaffin cells with a predominance of *CHGA, CHGB, PNMT*, *DBH* and *TH* (Figure 1D, Figures S2D and S3E and Table S1). Pathway analyses confirmed high enrichment of glutamatergic and cholinergic synapse pathways (Figure 2D).

### 2.3 Additional cell types of the adrenal microenvironment

As displayed in Figure 1, we detected five other cell populations as part of the normal adrenal glands’ microenvironment.

#### 2.3.1 Myeloid cells

Within the myeloid cells, we observed a large population of macrophages, mostly polarised into alternatively activated (M2) macrophages with high expression of *MRC1* (also known as *CD206*) and *CD163* together with *MSR1* (also known as *CD204*) (Figure 1D, Figure S2E and Table S1). Other highly expressed genes were *SRGN*, a proteoglycan that is typically expressed in myeloid and lymphoid cells, *CSF1R*, *CD86* and *CD14* (Figure S2E).

#### 2.3.2 Lymphoid cells

Lymphoid cells showed typical T cell markers, such as *IL7R* and *CD96* (Figure 1D and Table S1), as well as *CD247* (part of the T‐cell receptor‐CD3 complex), *RUNX3* and *ITK* (involved in T cell differentiation), *PRF1* and *GZMA* (indicators of granule mediated cytotoxic activity of natural killer and cytotoxic T cells) (Figure S2F and Table S1). Of note, only a few markers for B cells were found, for example, *IKZF3* (involved in the regulation of B lymphocyte proliferation and differentiation) (Figure S2F and Table S1).

#### 2.3.3 Fibroblasts and connective tissue cells

The fibroblasts and connective tissue cluster were characterised by cells that highly expressed collagen markers, such as *COL1A2* and *COL4A1*, and markers of the extracellular matrix, such as *MGP, FBLN1*, *LAMA2* and *CCN1* (Figure 1D, Figure S2G and Table S1). Using IHC, we confirmed the protein expression of COL1A2 in the adrenal capsule and MGP in the extracellular matrix and fibroblasts (Figure S3F). Pathway analyses validated the satellite cluster annotations, revealing enrichment of cluster‐specific gene sets, such as phagosome and NF‐kappa B signalling (FE > 4 for both pathways) for myeloid cells, Th1 and Th2 cell differentiation (FE > 5) and B and T cell receptor signalling pathway (FE > 4) for lymphoid cells, and focal adhesion (FE > 5) and extracellular matrix‐receptor interaction (FE > 4) for fibroblasts and connective tissue (Figure 2E–G).

#### 2.3.4 Vascular endothelial cells

**FIGURE 3:**
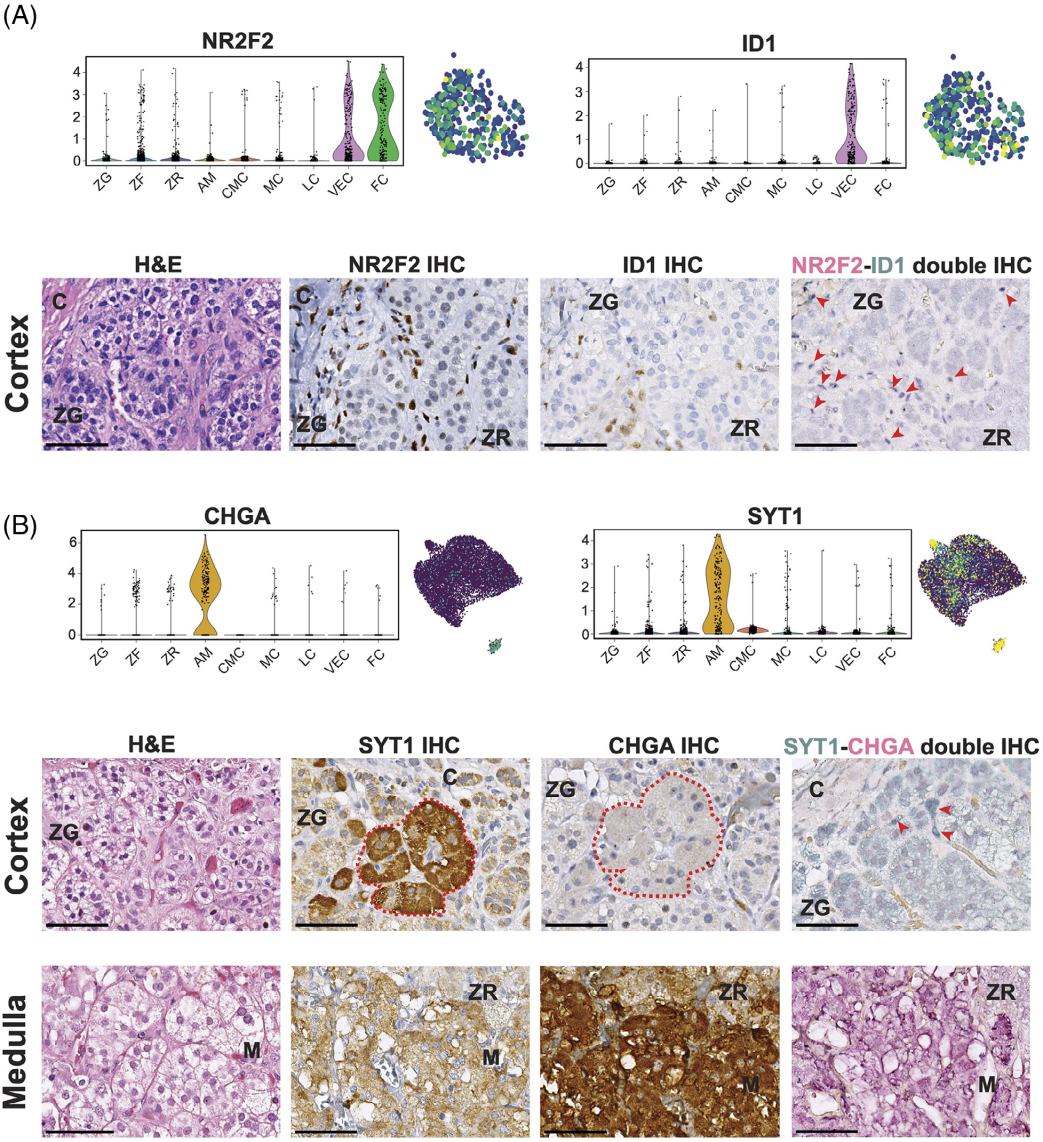
**New cell populations in adult human normal adrenal glands**. (A) Expression of *NR2F2* and *ID1* in the vascular‐endothelial cells (VEC) population at single‐nuclei transcriptomic level reported as a violin plot comparing the different clusters of the normal adrenal gland and as UMAP (Uniform Manifold Approximation and Projection). Immunohistochemistry (IHC) analysis revealed sparse cells with positive nuclear staining of NR2F2 or ID1, where ID1^+^ cells were rarer than NR2F2^+^ cells. These cells – although rare – were mostly located at the subcapsular level, while sparse cells were also found in the inner cortex. Nuclei of cells NR2F2^+^‐ID1^+^ were stained in dark blue at double immunostaining (sum of fuchsin‐red + blue‐green colours, indicated by red arrows) and were mostly located under the capsule. NR2F2^+^‐ID1^+^ nuclei are indicated with arrows. (B) Expression of *SYT1* and *CHGA* in the cortical‐neuroendocrine cells (CNC) at single‐nuclei transcriptomic level reported as a violin plot comparing the different clusters of the normal adrenal gland and as UMAP. In IHC, SYT1^+^ cells were grouped, forming a cluster in the subcapsular region (highlighted in red dashed line) in some of the evaluated samples. These cells were negative for CHGA, as confirmed also by the double immunostaining, where SYT1 strong positive cells (starker green‐blue colour, indicated by arrows) are found. On the contrary, medullary cells showed a strong staining for the CHGA and only a low staining for SYT1. Pictures of the same area of FFPE slides belonging to the same tissue were reevaluated for the different staining for each cell population. All images were acquired by Leica Aperio Versa brightfield scanning microscope (Leica, Germany). Scale bar: 100 µm. Abbreviation: C, capsule; H&E, haematoxylin and eosin staining; M, medulla; ZF, zona fasciculata; ZG, zona glomerulosa; ZR, zona reticularis.

The next cell type we identified expressed well‐known endothelial genes, such as *FLT1* (also known as *VEGFR1*)*, KDR* (also known as *VEGFR2*), *TEK* and *ENTPD1*, and termed this population “vascular‐endothelial cells” (VEC). These cells expressed also several genes associated with cell proliferation and differentiation, including *ETS1*, *ETS2*, *INSR* and *DNASE1L3* (Figure S2H and Table S1). Moreover, they are characterized by a low expression of genes associated with steroidogenesis, i.e., *NR5A1* and *STAR*. In addition, these cells overexpress *NOTCH1*, a known marker of adrenal cortical progenitors^38^ (involved in cell fate specification, differentiation, proliferation, and survival) and its target gene *HES1* (responsible for the regulation of genome‐wide glucocorticoid signalling^39^) as well as *ID1*, a known marker of mesenchymal stem cells (Figure S2H and Figure 3A).^40^, ^41^ Moreover, a distinct expression of *NR2F2* (also known as *COUP‐TFII*), a potential marker of mesenchymal cells in different adrenal models,^42^, ^43^, ^44^ was detected within this cluster as well as in the fibroblasts and connective tissue cluster (Figure 3A). *NR2F2* expression in VEC and FC populations aligns with findings from a previous study, which showed its presence in both the adrenal capsule and cortex by immunofluorescence.^45^ Here, the authors also showed that some of the NR2F2^+^ cells within the capsule co‐expressed GLI1, suggesting that these double‐positive cells could represent potential adrenocortical progenitors. In our dataset of adult adrenal glands, as well as in another recent study on human adrenals,^25^ no expression of *GLI1* was found. To validate the expression of NR2F2 mesenchymal cells in adult adrenal glands, we investigated the expression of NR2F2 at the protein level by IHC. We also tested the protein expression of ID1, known for its implication in adrenal tumorigenesis in dogs.^46^ NR2F2 staining was observed primarily in sparsely distributed endothelial cells mainly located at the subcapsular level, whereas few additional NR2F2^+^ cells were detected in the inner cortex (Figure 3A). ID1 nuclei staining showed a similar distribution, although a lower number of positive cells was observed compared to NR2F2^+^ cells (Figure 3A). The median number of ID1^+^ cells within each tissue was 1.3% (range: 0.5%−1.7%). The distribution of ID1^+^ cells was also validated by RNAscope, which showed sparse or grouped *ID1^+^* cells usually located around the adrenocortical cells of the ZG and ZF (Figure S4A). Via double immunostaining, we found sparse NR2F2^+^‐ID1^+^ cells mostly localized at the capsular/subcapsular region (Figure 3A). NR2F2^+^‐ID1^+^ cells had also a different distribution compared to the “classical” endothelial cells, which were stained for ENTPD1 and localized within the entire cortex (Figure S3G). Pathway analysis within the VEC population revealed significant enrichment of adherens junction (FE > 5) together with different signalling pathways, such as mitogen‐activated protein kinase (MAPK, FE > 3), Wnt pathway (FE > 2) and signalling regulating the pluripotency of stem cells (FE > 2) (Figure 2H). In line with the pathway analysis, the proliferative marker Ki67 was also positively expressed in very few endothelial cells (median < 1%; Figure S4B). However, immunofluorescence (IF) showed that Ki67^+^ cells were not the same as NR2F2^+^ cells (Figure S4C). Taking these results into account, we have hypothesized that different subpopulations of endothelial cells are present within the cortex of NAGs.

#### 2.3.5 Cortical‐neuroendocrine cells

Within the main adrenal cortex cluster, we identified a potential new rare population of cells with sympathoadrenal lineage characteristics in addition to adrenocortical features. This population, which we referred to as “cortical‐neuroendocrine cells” (CNC), expressed essential steroidogenesis enzymes, including *NR5A1* (also known as *SF1*)*, STAR*, and *CYP17A1* (Figure S5A) together with high expression of genes associated with neurotransmission, such as *SYT1, NRG1*, *NRXN3, GRIK1* and *CADPS* (Figure 1B, Figures S2I, S4B and Table S1). These genes associated with neurotransmission were upregulated also in medullary cells. However, unlike mature medullary cells, CNC did not express genes like *CHGA, CHGB*, *TH*, *PNMT* and *DBH*. Gene enrichment analysis supported the neuroendocrine nature of CNC, revealing sets of genes involved in cholinergic and glutamatergic synapse (FE > 7), glutamatergic synapse (FE > 5), and axon guidance (FE > 4) (Figure 2I). The evaluation of CNC was further examined through IHC, with consecutive sections used for SYT1 and CHGA staining (Figure 3B). As per the transcriptomic data, the cortex displayed relatively low SYT1 staining (median score 118 (interquartile range 66–133), while the medulla exhibited moderate staining (median score 151 (119–164). However, high SYT1^+^ staining was observed in rare cells within the cortex (Figure S6A,B), which were organized as sparse groups of cells in the subcapsular region (maximum number of two groups per slide within a median evaluated cortex area of 111 mm^2^ (64.5–170.9)) in six out of the 16 evaluated NAGs (37.5%) (Figure 3B (SYT1^+^ niche highlighted in red) and Figure S6C–H). SYT1^+^ cells presented a median IHC score of 181 (66–205), which was higher compared to the overall score in the cortex and medullary cells (*p *< 0.0001 and *p *= 0.03, respectively, Figure S6I). The median percentage of adrenocortical SYT1^+^ cells (considering only those with high SYT1 staining) within the cortex was 0.3% (interquartile range 0.20%−0.95%) per tissue. In line with snRNA‐seq data, these cells did not show CHGA expression in the double SYT1‐CHGA staining (Figure 3B).^47^ Moreover, IF double staining showed the presence of rare CYP17A1^+^‐ SYT1^+^ cells sparse within the cortex in 37% of evaluated tissues (Figure S7). Overall, these data indicate that the CNC are different from both cortex and medullary cells.

### 2.4 Adrenal cortex zonation by spatial transcriptomics in NAG

**FIGURE 4:**
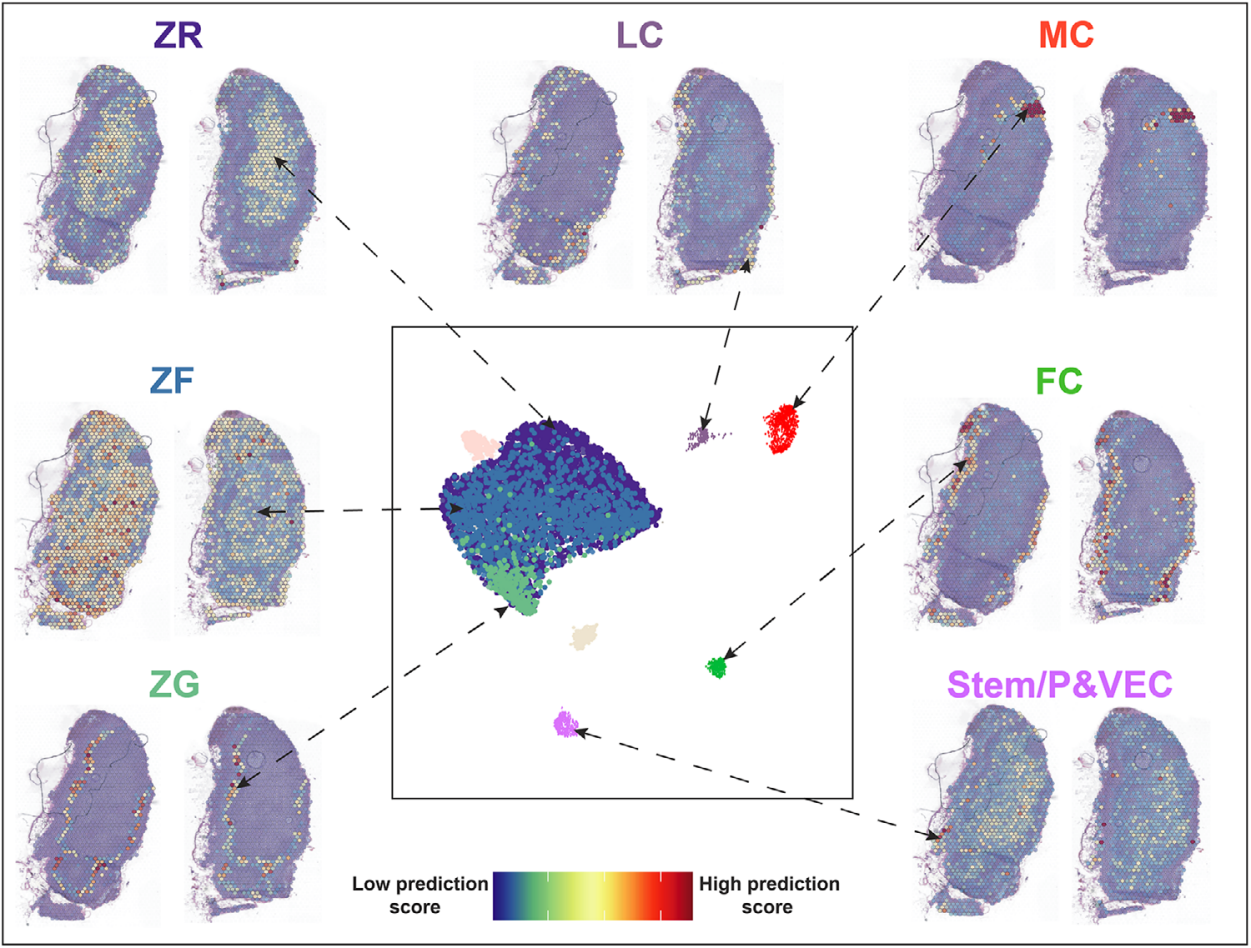
**Validation of adrenal zonation by spatial transcriptomics in adult human normal adrenal glands**. A pairwise (cell‐to‐spot) score is calculated within the label transfer framework in Seurat (V3) and projected onto sections. Two sections are represented for each mapped cluster. Medulla and cortical‐neuroendocrine cells (CNC) were rendered transparent as the label transfer did not detect the presence of these cell types in the Visium assay. Abbreviations: FC, fibroblasts & connective tissue; LC, lymphoid cells; MC, myeloid cells; VEC, vascular‐endothelial cells; ZF, zona fasciculata; ZG, zona glomerulosa; ZR, zona reticularis.

Cellular and tissue assignments of genes based on snRNA‐seq data were validated by spatial transcriptomics using the Visium assay in two consecutive adrenal gland sections deriving from the patient sample #NAG‐7. As we further focused in this study on the adrenal cortex, the medulla was not part of the analysed tissue sections. To identify each zone, we transferred the cell type annotation from the microdroplet‐based snRNA‐seq into the Visium dataset using a cell‐to‐spot correlation framework (Figure 4). Mapping of the adrenal cortex zone markers confirmed the snRNA‐seq dataset. ZG appeared as a thin line of cells towards the outer cortex, while the distribution of spots with high ZF scores was rather ubiquitous. Moreover, spots with higher ZR scores seemed to be located closer towards the centre of the tissue section, although a substantial overlap between ZF and ZR was observed (Figure 4 and Figure S8). It is important to note that certain genes identified from the snRNA‐seq data (e.g., *DACH1* for ZG and *CYB5A* for ZR) displayed weak or no expression in the Visium dataset. This discrepancy could be attributed to either the lower RNA capture efficiency of Visium compared to single‐nuclei assays or the possibility of specific zones being underrepresented in the selected tissue sections. Regarding the satellite clusters, the label transfer predicted similar mapping for fibroblasts and connective tissue and lymphoid cells to the region surrounding the cortex, although the latter displayed a low overall mapping score (Figure 4). Myeloid cells showed a specific expression mostly around the small blood vessels within the adrenal cortex (Figure 4). Finally, VEC mapped with a high prediction score in the outer cortex, specifically localized in a subcapsular niche, while a medium to low prediction score was observed in the inner cortex (Figure 4).

### 2.5 Pseudotime analysis supports the centripetal maintenance dynamics within the adrenal cortex

**FIGURE 5:**
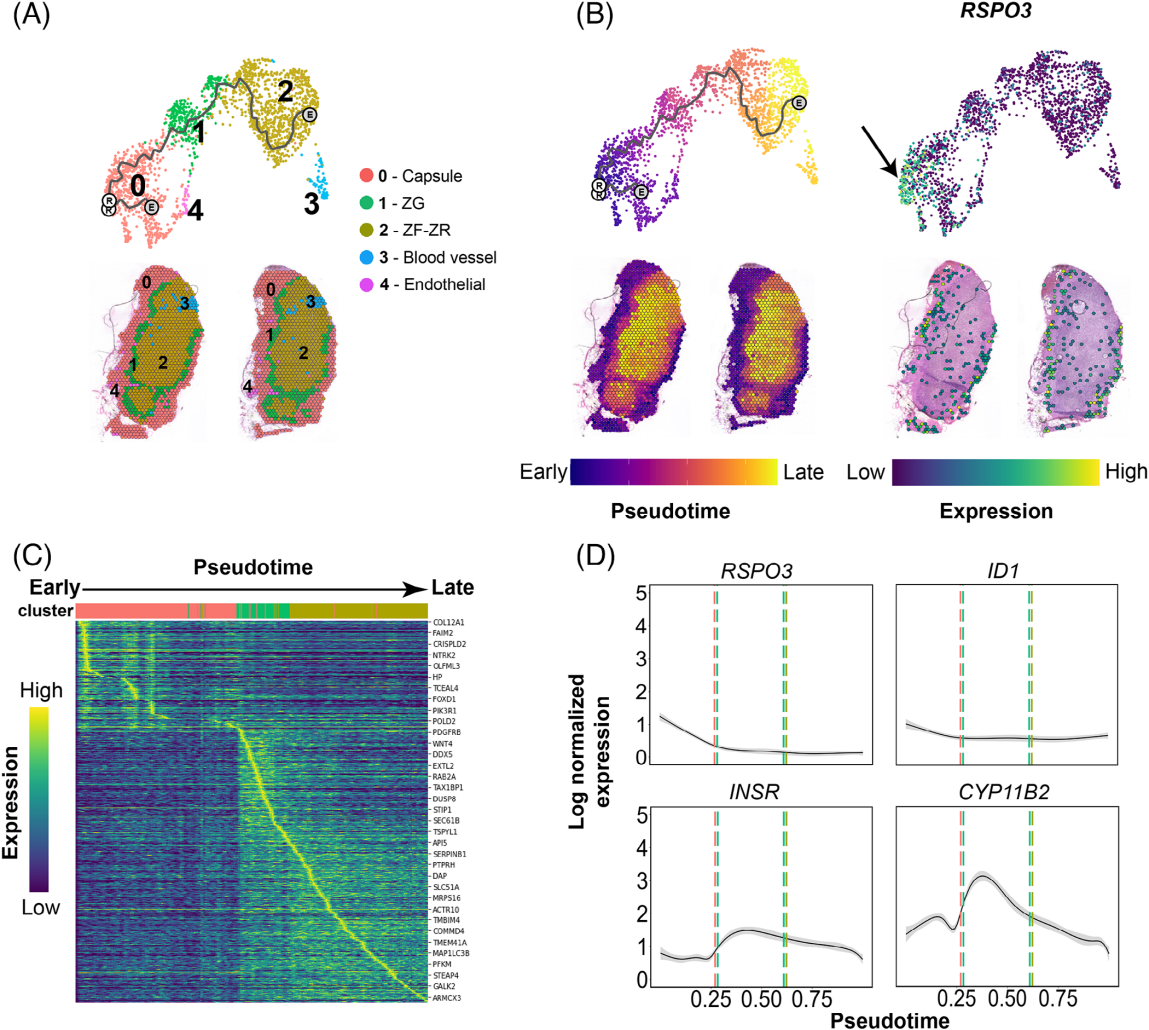
**Trajectory analysis of spatial transcriptomics data in adult human normal adrenal glands**. (A) Top: Integrated UMAP (Uniform Manifold Approximation and Projection) from both tissue sections analysed using the Visium assay (10x Genomics). The trajectory was visualised as a black line. Bottom: clusters identified in the integrated object transferred to the tissue (Visium) sections. (B) Pseudotime estimation both for the UMAP and the tissue sections: white circles represent the root node (chosen based on *RSPO3* expression, indicated with a black arrow (right panel)). (C) Heatmap of 829 differentially expressed genes across pseudotime: cells were ordered by pseudotime (columns), and genes were ordered by expression and pseudotime (rows). The top genes are highlighted. (D) Expression variation of *RSPO3, ID1, INSR* and *CYP11B2* over pseudotime: smoothed lines were generated based on the scatter profile of each gene (95% confidence interval displayed in grey around the line). Vertical lines represent the transition zones from 0‐Capsule (red) to 1‐ZG (green) and 1‐ZG to 2‐ZF‐ZR (ochre).

Next, we aimed to understand the developmental relationship between the observed cell clusters within the adrenal cortex. To predict a differentiation trajectory, we performed pseudotime analysis^48^ using the spatial transcriptomics data. The integration of the two NAG replicates resulted in 5 distinct Louvain clusters (0 = Capsule, 1 = ZG, 2 = ZF‐ZR, 3 = blood vessel, 4 = endothelial) that we annotated based on the results of the label transfer (Figure 5A). We chose cells expressing RSPO3 as the root node for pseudotime computation. RSPO3 is a widely recognized marker for capsule adrenocortical progenitors in mouse adrenal glands,^34^, ^45^ and it was found to be expressed by a distinct subset of cells within the capsule (Figure 5B). The resulting unsupervised lineage trajectory of the adrenal cortex started with capsular cells, proceeded through the ZG, and ended in the ZF‐ZR cells, indicating a centripetal development of the adrenal gland.

Pseudotime analysis in DEGs of the three main clusters (capsule, ZG, and ZF‐ZR) was performed to investigate potential changes in gene expression dynamics along the trajectory (Figure 5C). A total of 829 genes significantly varied across pseudotime. Among them, *RSPO3* was strongly expressed in capsular cells at an early pseudotime stage and weakly expressed within the inner‐zones representing later cell types (Figure 5D). A similar pattern of cellular development was observed for *ID1*. *INSR* gene expression was specifically switched on in ZG (early time point in development) and its expression diminished throughout the inner cortex (later time points) (Figure 5D). The selective activation of these genes at the early stage confirmed the progenitor‐like nature of the VEC population found in the snRNA‐seq analysis. Among the steroidogenic genes, C*YP11B2* was first activated in the early ZG cells and significantly decreased throughout the ZF‐ZR (Figure 5D). We also noted a slight increase in *CXCL12* expression, associated with mesenchymal cells in the fetal adrenal cortex,^25^ in the transition zone between the capsule and ZG. Additionally, the gene encoding its receptor, *CXCR4*, exhibited increased expression in the late ZG cells and across the populations of the inner cortex (Supplemental Figure 9). A similar expression to *CXCR4* was observed for *PRKAR1A* (cAMP/PKA pathway). The transcriptional coactivator *CITED2*, which also plays a role in the development of the adrenal glands by stimulating the expression of *NR5A1*,^49^ showed a first peak in early capsular cells and a second one in the ZG (Figure S9).

Moreover, three key signalling systems, including the Wnt/ β‐catenin, the SHH and the FGF pathways, were investigated by pseudotime analysis (Figure S9). Among the Wnt/β‐catenin pathway‐related genes, *SFRP1*, *SFRP2* and *SFRP4* were found to be expressed in early or late capsular cells, but their expression decreased throughout the remaining cell subpopulations. *WNT4* was expressed in ZG only. Other genes such as *CTNNA1* or *CTNNA1L* were specifically switched on in the late ZG subpopulation and continued to be mildly expressed in the ZF‐ZR zones (Figure S9). Within the SHH pathway, *ETS2*, a transcription factor involved in tissue development and cancer progression,^50^ was slightly more expressed in the capsular zone (Figure S9). *SHH* was relatively highly expressed in ZG and decreased towards the inner zones of the cortex. Other genes like *NRCAM* and *NRP1* had high expression in the ZG cells and were quite constant throughout the inner cortex, whereas the expression of *VEGFA*, which is a downstream target of SHH, progressively increased from the outer to the inner part of the adrenal cortex (Figure S9). Different genes of the FGF pathway were distributed between the capsule and ZG area. *FOS* and *JUN* followed a high expression trend decreasing from the capsule towards the inner cortex (Figure S9). These two genes belong to the Fos and Jun gene family, which encode protein subunits collectively constituting the transcription factor complex, known as activating protein‐1, which plays a pivotal role in regulating numerous biological processes, including cell proliferation and differentiation.^51^ A similar distribution, although not so high, was found also for *FGFR1*. On the other hand, genes like *FGFR2*, *AKT1*, *NCAM1* and *HGF* where more expressed in ZG and in the inner cortex (Figure S9). The module score analysis displayed an early activation of the Wnt/β‐catenin and FGF pathways, which then decreased towards the inner cortex, whereas SHH exhibited stable basal activity throughout pseudotime (Figure S9).

Of note, *DLK1* (Delta‐like homologue 1), a member of the Notch signalling pathway, was found to be highly expressed in the ZG to ZF‐ZR transition zone (Figure S10A,B—yellow box). Interestingly, *DLK1* expression visualized on the adrenal sections revealed a similar pattern described by Hadjidemetriou et al.,^52^ that is, as “DLK1‐expressing cell clusters” (Figure S10C, indicated by the yellow box).

### 2.6 Integration of NAG and ACA snRNA‐seq data reveals adenomas‐specific cells

The NAG snRNA‐seq atlas was used as a reference to evaluate cell type heterogeneity in ACA samples (Table 2). 21,794 single‐nuclei transcriptomes from 12 snap‐frozen ACA samples (seven CPAs and five EIAs) were sequenced and integrated this data with 5154 highest‐scoring NAG nuclei.

**TABLE 2:** Overview of demographic, clinical and genetic details for the 12 adrenocortical adenomas evaluated by single nuclei RNA sequencing.

ID	Hormone pattern	Driver mutation	Sex/Age, years	Tumor size, cm	Cortisol after DST, nmol/L	LNSC, nmol/L	24h UFC, nmol/d	Basal ACTH, pmol/L	#of QC'ed nuclei	Mean Counts	Mean Features per nucleus
**EIA‐1**	Endocrine inactive	*CTNNB1*	F/67	3, 4	n.a.	n.a.	84	1,4	1903	447	366
**EIA‐2**	Endocrine inactive	no	F/82	2, 8	n.a.	n.a.	n.a.	n.a.	1860	647	516
**EIA‐3**	Endocrine inactive	*CTNNB1*	F/62	4, 7	35, 9	n.a.	65	1,3	2208	640	528
**EIA‐4**	Endocrine inactive	*CTNNB1*	F/59	5, 3	35, 9	n.a.	220	n.a.	1760	922	719
**EIA‐5**	Endocrine inactive	*CTNNB1*	F/56	6, 0	n.a.	n.a.	146	2, 4	1537	577	434
**CPA‐1**	Overt Cushing	*PRKACA*	F/60	3, 5	96, 6	37, 2	745	1, 9	2415	762	596
**CPA‐2**	Overt Cushing	*PRKACA*	F/29	3, 3	n.a.	24, 8	4889	< 1.1	2448	623	484
**CPA‐3**	Overt Cushing + androgens	*CTNNB1*	M/64	7, 0	74, 5	12, 7	249	2,9	1377	531	407
**CPA‐4**	Overt Cushing	*CTNNB1*	F/57	6, 0	689, 7	31, 4	1709	< 1.1	1764	540	424
**CPA‐5**	Overt Cushing	no	F/43	2, 5	761, 5	49, 4	713	< 1.1	1702	440	353
**CPA‐6**	Overt Cushing	no	F/59	3, 5	201, 4	4, 7	410	< 1.1	1642	555	438
**CPA‐7**	Overt Cushing	*GNAS*	F/23	3, 2	642, 8	39, 2	1670	< 1.1	2335	485	396

Overt Cushing syndrome was defined as the presence of typical clinical signs and at least two pathological screening tests and suppressed plasma ACTH (details see Methods).

Abbreviations: ACTH, adrenocorticotropic hormone; CPA, cortisol‐producing adenoma; *CTNNB1*, β‐catenin; DST, overnight 1 mg dexamethasone suppression test; EIA, endocrine‐inactive tumour; F, female; GNAS, stimulatory G‐protein α subunit; LNSC, late‐night salivary cortisol; M, male; n.a., not available; PRKACA, catalytic subunit α of protein kinase A; 24 h UFC, 24‐h urinary free cortisol; #, number.

**FIGURE 6:**
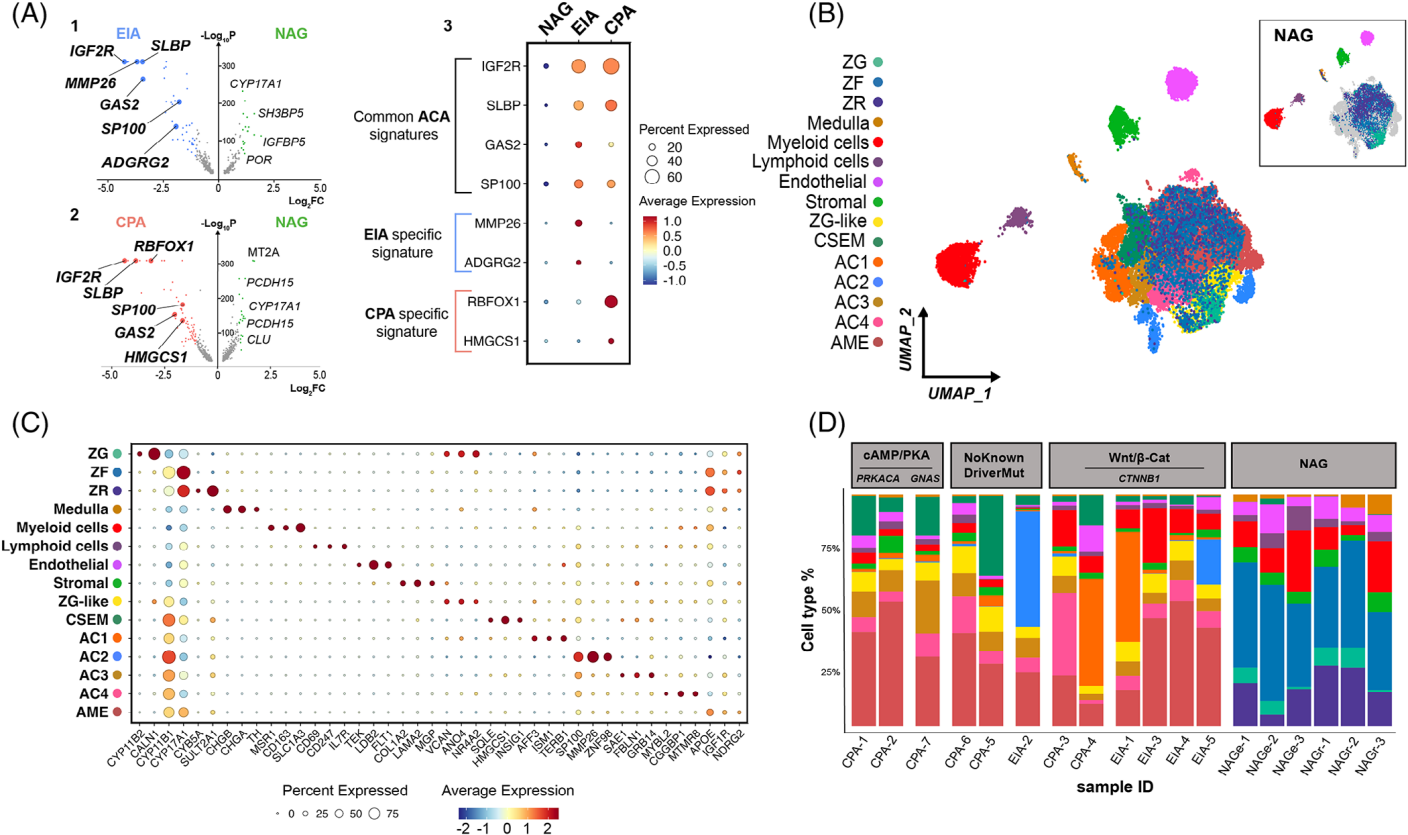
**Identification of adenoma‐specific clusters in adrenocortical adenoma**. (A) 1. Volcano plot depicting differentially expressed genes between normal adrenal glands (NAG) and endocrine‐inactive adenomas (EIA). Genes characteristic of the EIA subtype are written in bold. The x‐axis represents fold change (Log_2_FC) and the y‐axis represents P values (‐Log_10_P). 2. Volcano plot depicting differentially expressed genes between NAG and cortisol‐producing adenomas (CPA). Genes characteristic of the CPA subtype are written in bold. 3. Dot plots showing log normalized expression values of common signatures across the three subtypes (NAG, EIA, CPA). (B) UMAP (Uniform Manifold Approximation and Projection) representation of the 18 integrated samples (NAG, *n* = 6; CPA, *n* = 7; EIA, *n* = 5) coloured by cluster identity. The UMAP could be divided into two groups: the “central cluster” containing previously identified cortex cell types (ZG, ZF and ZR) as well as ZG‐like, adenoma‐specific clusters (AC1, AC2, AC3 and AC4), Cholesterol‐and steroid‐enriched metabolism (CSEM), and adenoma microenvironment (AME) clusters, and the “satellite” populations, which are medulla, myeloid cells, lymphoid cells, endothelial and stromal. NAG cell type distribution in the UMAP is represented within the top right box. C. Scaled average expression of selected markers for each cluster (dot size represents the percentage of cells in each cluster expressing the marker). (D) Subtype and sample‐specific composition coloured by cluster identity: samples (x‐axis) are ordered by mutational signatures.

The differential expression analyses indicated that several genes involved in cell signalling, tissue remodelling, cell replication and RNA transcription were significantly upregulated in ACAs compared with NAGs (Figure 6A‐1,2). Compared to NAG, genes like *IGF2R* (average fold‐change for gene expression, avg‐log2FC > 4.2, *p *< 1e‐6 in both EIA and CPA), *SLBP* (avg‐log2FC > 3.4, *p *< 1e‐6 in both EIA and CPA), *GAS2* (avg‐log2FC > 3.4, *p *= 1.04e‐272 in EIA and avg‐log2FC > 2.0, *p *= 8.85e‐153 in CPA), and *SP100* (avg‐log2FC > 1.8, *p *= 2.45e‐208 in EIA and avg‐log2FC > 1.6, *p *= 9.33e‐184 in CPA) were strongly expressed in both EIA and CPA (Figure 6A‐3). Genes like *MMP26* (avg‐log2FC > 3.6, *p *< 1e‐6) and *ADGRG2* (avg‐log2FC > 1.5, *p *= 2.27e‐138) were strongly expressed in EIAs, whereas *RBFOX1* (avg‐log2FC > 3.1, *p *< 1e‐6) and *HMGCS1* (avg‐log2FC > 1.6, *p *= 4.51e‐134) were predominantly expressed in CPAs (Figure 6A‐4).

The integration of NAG and ACA revealed a primary central cluster and 5 satellite clusters comprised of myeloid, lymphoid, endothelial, stromal (mainly composed of fibroblasts and connective tissue) and medullary cells (Figure 6B). The central cluster showed a complex organization including the three cortex‐specific zones, for example, ZG, ZF and ZR, and seven other cell populations (Figure 6B,C). The “cholesterol‐and steroid‐enriched metabolism” (CSEM) population was also found in a very small percentage of NAGs (∼1%). The six remaining cell populations were specifically present in ACAs and were named “adenoma microenvironment” (AME), “ZG‐like”, and “adenoma‐specific clusters” (AC1‐4). The top 100 DEGs defining the individual clusters of the NAG‐ACA integration are listed in Table S2. Of note, *CYP17A1* and *CYP11B1* were ubiquitously expressed at low to medium levels across all clusters (Figure 6C), confirming the adrenocortical nature of these cells.

### 2.7 ACA‐specific clusters distribution according to subtype and mutational status

We further explored the cell type composition across the 18 samples by grouping our samples according to tissue entity (CPA, EIA or NAG) and mutational status—available from previous next‐generation DNA or Sanger sequencing of bulk tumour tissues (no known driver mutation, *CTNNB1* mutations, and *PRKACA/GNAS* mutations) (Figure 6D).

The ZG‐like and the AME clusters showed a homogeneous distribution, without significant differences among the samples (Figure 6D). Within the ZG‐like cluster, common ZG markers, such as *DACH1*, *ANO4* and *NR4A2* (also known as *NURR1*) were dominantly expressed (Figure S11A), indicating the presence of normal adrenocortical cells in ACAs. However, unlike ZG, cells within the ZG‐like cluster were characterised by a very low expression of *CYP11B2* (avg‐log2FC > −2, *p *= 8.69e‐36), *CALN1* (avg‐log2FC > −2.3, *p *= 9.60e‐82) and *MC2R* (avg‐log2FC > −1.9, *p *= 1.17e‐31), and high expression of *CYP11B1* (avg‐log2FC > 1.2, *p *= 2.05e‐16), *IGF2R* (avg‐log2FC > 4.4, *p *= 3.79e‐78), and *GAS2* (avg‐log2FC > 2.4, *p *= .82e‐12). Pathway analyses revealed the enrichment of several pathways involved in cell signalling, such as protein phosphorylation (FE > 6) and calcium signalling (FE > 3) (Figures S11B and S12).

Cells of the AME overexpressed genes like *APOE*, *ADIPOR2* and *RARRES2* are involved in the lipoprotein's metabolism, as well as *IGF1R* and *NDRG2* (Figure 6C and Figure S11A). *IGF1R* is a transmembrane tyrosine kinase receptor of the IGF family that activates a mitogenic signalling pathway, stimulating cell proliferation, division, and translation, and inhibiting apoptosis.^53^ *NDRG2* regulates the Wnt signalling pathway by modulating *CTNNB1*‐target genes.^54^ Of note, key steroidogenesis markers including *NR5A1* and *CYP17A1* were heavily expressed in this cluster (Figure S11A). Pathway analyses revealed enrichment of several pathways involved in cell signalling, such as AMPK (FE > 4) and insulin signalling pathways (FE > 3), cholesterol trafficking (FE > 4), and pathways associated with different tight, focal and adherens junctions (FE > 2) (Figures S11B and S12).

**FIGURE 7:**
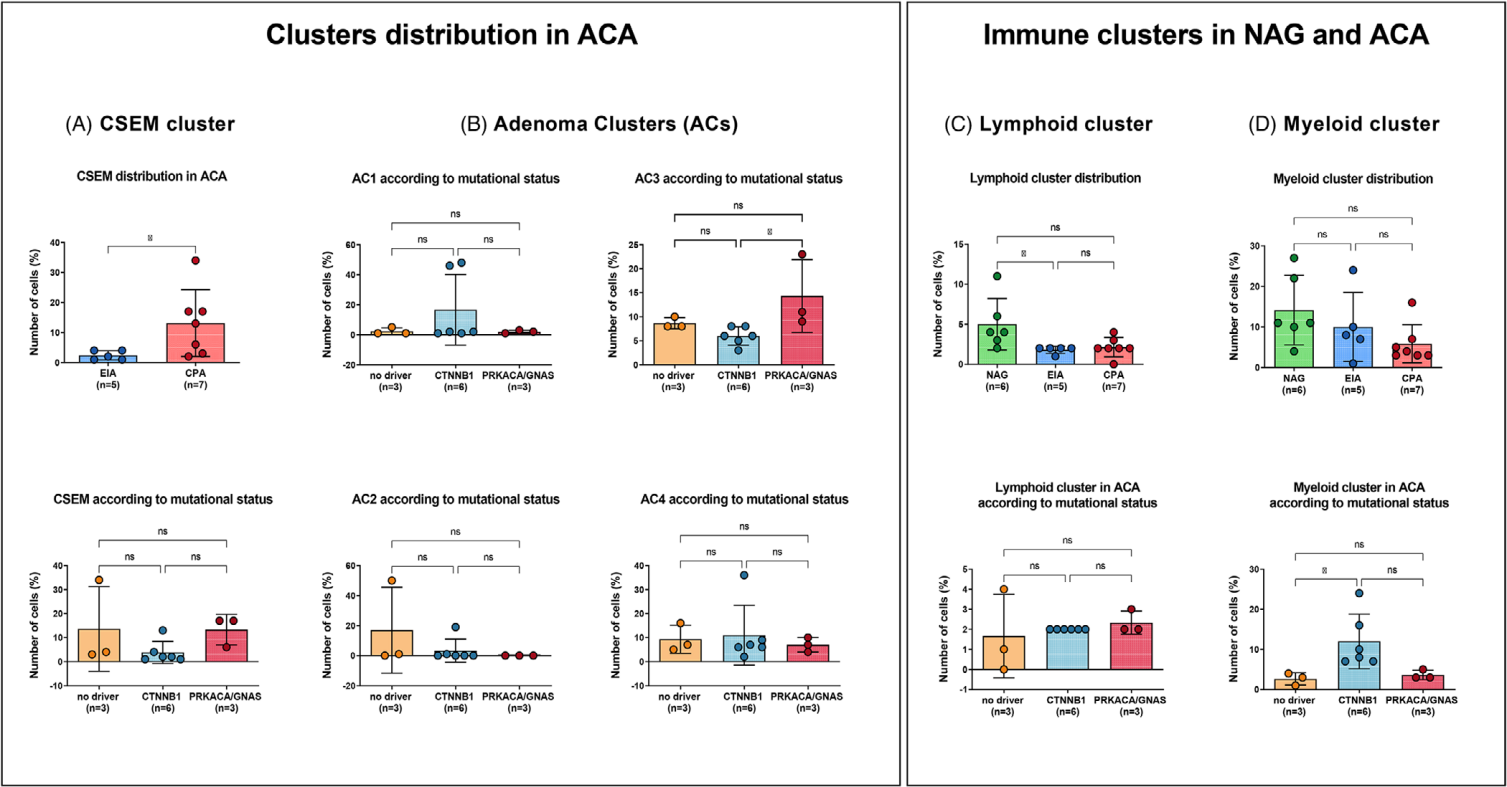
**Cluster distribution in ACA samples across subtype and mutational status**. Distribution of (A) cholesterol‐and steroid‐enriched metabolism (CSEM) cluster in cortisol‐producing adenomas (CPA) versus endocrine‐inactive adenomas (EIA; upper panel) and across mutational status (lower panel); (B) Adenoma clusters (AC1‐4) across mutational status; (C) Lymphoid cluster across subtypes (upper panel) and mutational status (lower panel); (D) Myeloid cluster across subtypes (upper panel) and mutational status (lower panel). Significant cluster enrichments are encircled in red.

The CSEM cluster was significantly over‐represented in the CPA samples compared to EIAs (*p *= 0.03; Figure 7A), with high expression of genes related to cholesterol metabolism, including *HMGCS1*, *SQLE*, and *INSIG1*, but also *KCND2*and *POLE2* (Supplemental Figure 11A) involved in DNA repair and replication. The pathway enrichment analyses confirmed this finding, highlighting a significant enrichment of various pathways related to cholesterol metabolism and steroidogenesis, such as cholesterol and steroid biosynthesis (FE > 9), pathways involved in ubiquitination processes, including protein ubiquitination (FE > 5), and cellular response to oxidative stress (FE > 2.5) (Figures S11B and S12).

The remaining clusters presented a slightly different distribution considering tumour subtypes and mutational status. Specifically, AC1 was mostly found in two *CTNNB1*‐mutated samples, one EIA (EIA‐1, ∼48%) and one CPA (CPA‐4, ∼46%) (Figures 6D and 7B), with a high expression of the β‐catenin target genes *LEF1*, *AFF3*, and *ISM1* (Figure 6C, Figure S11A and Table S2). Both *AFF3* and *ISM1* are known to be overexpressed in benign and malignant adrenocortical tumours with *CTNNB1* mutation.^55^, ^56^, ^57^ Also, we found a high expression of *FTO*, an N6‐methyladenosine demethylase that promotes cell proliferation, migration, and chemo‐radiotherapy resistance by targeting *CTNNB1* in different cancer types^58^, ^59^ (Figure S11A). In addition, other genes involved in cell proliferation, including *GAS2* and *TERB1*, or *SHOC1*, involved in the formation of meiotic crossovers, and long non‐coding RNAs, such as *CASC16*, were found to be highly expressed within this cluster (Figure S11A). The enrichment analysis revealed a very high expression of different pathways involved in spliceosome (FE > 10), cell proliferation and tissue development (FE > 3), and extracellular cell‐matrix adhesion (FE > 5) (Figures S11B and S12).

AC2 cluster was more abundant in two EIA samples (EIA‐2 and EIA‐5) (Figure 6D) without correlation with the mutational status (Figure 7B). The AC2 cluster was characterised by an overexpression of genes like *MMP26*, *ZNF98* and *SP100* associated with tumour promotion (Figure 6C and Figure S11A). Enrichment analysis showed also for AC2 a high expression of spliceosome pathway (FE > 6) and ubiquitin‐related pathways, including SUMOylation (FE > 5) and cell cycle (FE > 2) (Figures S11B and S12).

In contrast, AC3 was significantly over‐represented in CPAs with *PRKACA/GNAS* mutation compared to *CTNNB1‐*mutated samples (*p *= 0.02) (Figures 6D and 7B). Particularly, AC3 was very abundant in the single CPA with *GNAS* mutation (CPA‐7, ∼23%). Of note, we observed a high expression of *PDE8A* within this cluster (Figure S11A). Also, a high expression of *SAE1*, associated with SUMOylation, *GRB14* and *FBLN1*, associated with cell growth and extracellular matrix, respectively, was found (Figure 6C and Figure S11A). Enrichment analyses confirmed a high enrichment in genes of the SUMOylation pathway (FE > 4), cell adhesion molecular binding (FE > 3), but also spliceosome (FE > 2) (Figures S11B and S12). Interestingly, AC1‐3 showed a high enrichment of elements associated with spliceosome, which were represented at very high levels in AC1 and decreased through AC2 and AC3.

Looking into the AC4 cluster, its distribution was similar among all tumour samples except for one CPA with *CTNNB1* mutation (CPA‐3), where it represented ∼36% of the cells (Figures 6D and 7B). Within this cluster, we found a high expression of the proto‐oncogene *MYBL2*, as well as of *CGGBP1*, which regulates the cell cycle in cancer cells (Figure 6C and Figure S11A). Pathway analyses showed significant enrichment of several pathways involved in extracellular matrix, cell‐cell communication (FE > 2) or different types of cell junctions (FE > 2) and oxidative stress‐induced senescence (FE > 3) (Figures S11B and S12).

### 2.8 Immune clusters

We noted a reduction in the number of both lymphoid and myeloid cells when comparing NAGs to ACAs (Figure 7C,D). Particularly, the lymphoid cluster was predominantly enriched in NAGs compared to EIAs (*p *= 0.02) and by trend compared to CPAs (*p *= 0.09) (Figure 7C). No difference was observed among the ACAs according to the mutational status. Genes like *HLA‐A*, *HLA‐B*, *HLA‐C*, and *CCL5* followed a trend to decreased expression in CPA compared to NAG (avg‐log2FC > −1.3, *p *= 2.84e‐08 for *HLA‐A*, avg‐log2FC > −1.2, *p *= 6.55e‐07 for *HLA‐B*, avg‐log2FC > −1.7, *p *= 5.58e‐07 for *HLA‐C*, avg‐log2FC > −1.5, *p *= 1.05e‐06 for *CCL5*) (Figure S13A).

The myeloid cluster was similarly represented in NAGs when compared to EIAs, but less represented in CPA samples (*p *= 0.07) (Figure 7D). Interestingly, the distribution of the myeloid cluster among the ACA samples changed according to the mutational status. ACA samples with *CTNNB1* mutation revealed a higher abundance of myeloid cells compared to samples with no known driver (*p *= 0.03) and *PRKACA/GNAS* mutations (*p *= 0.08) (Figure 7D). Among differentially expressed genes (Figure S13B), *MSR1* was significantly downregulated in EIAs (avg‐log2FC > −1.2, *p *= 2.5e‐19) and CPAs (avg‐log2FC = −0.9, *p *= 4.49e‐12) compared to NAGs. Similarly, *SRGN* decreased in EIA (avg‐log2FC > −1.2, *p *= 5.24e‐27) and CPA (avg‐log2FC > −0.6, *p *= 3.57e‐11) compared to NAGs. Genes like *CSF1R* and *CD74* were less expressed in CPAs compared to both NAGs (avg‐log2FC > −0.9, *p *= 2.07e‐07 for *CSF1R* and avg‐log2FC −0.9, *p *= 1.87e‐10 for *CD74*) and EIA (avg‐log2FC > −1.0, *p *= 3.04e‐10 for *CSF1R* and avg‐log2FC > −1.3, *p *= 4.38e‐24 for *CD74*) (Figure S13B).

## 3 DISCUSSION

In this study, we present a comprehensive cell atlas of the normal adult human adrenal gland based on transcriptome analyses at the single‐nuclei level complemented by spatial RNA and protein expression results. Using this dataset, we were able to distinguish the characteristic zones of the adrenal cortex (ZG, ZF and ZR), as well as the adrenal medulla and capsule. In addition, we found different satellite clusters, including lymphoid, myeloid and vascular cells. Moreover, by comparing our single‐cell transcriptome data of the adult human normal adrenal glands to a cohort of adrenocortical adenomas, we revealed seven adenoma‐specific cell types that could be involved in adrenocortical tumorigenesis.

An intriguing observation of our study revolves around the identification of two potential new populations within the adult human NAG. The first population (vascular‐endothelial cells cluster) comprises a subset of endothelial cells distinguished by the co‐expression of NR2F2 and ID1. These cells represented approximately 1.3% of the adrenal cortex cell population. Immunohistochemistry showed that NR2F2^+^‐ID1^+^ cells are mostly located in the subcapsular region, although sparse NR2F2^+^‐ID1^+^ cells are also found within the inner part of the cortex. Together with *NR2F2* and *ID1* expression, which are both markers of mesenchymal cells and involved in cell differentiation,^45^, ^46^ this subpopulation of cells was also characterized by a high expression of genes associated with cell proliferation (including *ETS1*, *INSR*, *HES1*, *DNASE1L3* and *NOTCH1)*.^38^ Some of these genes were selectively activated at the early stages of pseudotime analysis. Further, the VEC cluster was also highly enriched of adherens junctions and vascular cells, which are essential in cell‐cell communication, cell renewal and differentiation.^60^, ^61^ However, IF analysis demonstrated that NR2F2^+^ cells were not expressing the proliferative marker Ki67. Taken together, our results suggest that NR2F2^+^‐ID1^+^ cells might represent a new population of endothelial cells located mostly underneath the capsule.

The second new cell population was indicated as “cortical‐neuroendocrine cells”, because of the expression of genes resembling neuronal‐like features, including *SYT1*, *NRG1*, and *NRXN3*, mimicking the medulla, and concomitant expression of markers associated with steroidogenesis. However, this population was void of markers of mature medullary cells such as *CHGA*. By performing immunostaining, we confirmed the presence of rare SYT1^+^‐CHGA^−^ cells in one‐third of the investigated samples. These cells were sparse within the cortex and sometimes organized in groups (or niches) in the subcapsular region. Some of these cells co‐expressed also CYP17A1 and SYT1, as demonstrated by IF. This finding suggests that these cells (or a subgroup of them) may possess a combined sympathoadrenal and steroidogenic expression profile, potentially playing a role in adult adrenal gland homeostasis.^45^, ^62^ However, further lineage tracing experiments are needed to better characterise this new subpopulation of cells.

The trajectory and pseudotime analyses allowed us to look more deeply into the transition zone between the capsular and subcapsular regions, where progenitor cells are mostly located. Here we showed, for the first time, selective activation of some members of the Wnt/β‐catenin pathway (i.e. *SFRP2* and *SFRP4* ^63^), which play an important role in adrenocortical renewal and differentiation,^64^ as well as in adrenocortical tumorigenesis.^14^, ^65^, ^66^ The FGF pathway was also activated early in pseudotime, supporting its role in adrenal development and homeostasis.^67^ Particularly, we have found a pronounced capsular expression of *FOS* and *JUN* encoding the subunits of the transcription factor activating protein‐1, which are known to play a role in steroidogenesis by regulating *NR5A1* (*SF‐1*) and *STAR*.^68^, ^69^ Another gene regulating the expression of *NR5A1*, and therefore of adrenal development, is *CITED2*.^49^, ^70^ This gene was selectively activated in the ZG in the pseudotime analysis. Of note, *CITED2* is regulated by *FGF2* in adrenocortical cells,^71^ again underlining the role of the FGF pathway in adrenal homeostasis. A relatively high expression in the ZG was found also for *SHH*, which is involved in the self‐renewing process for the ZG and ZF cells in animal models.^62^ It has been postulated that SHH and FGF pathways interact with each other in the adrenal homeostasis.^62^ The pseudotime analysis showed also an early activation of the *CXCL12* and a later activation of *CXCR4* in the ZG‐inner cortex, supporting the recent finding that the CXCL12/CXCR4 ligand‐receptor signalling system may play a role in the human adrenal development and homeostasis.^25^

Intriguingly, *DLK1*, a gene known to be implicated in rat adrenocortical zonation and remodelling^72^ and in human adrenocortical carcinomas,^30^ was found to be highly expressed in the ZG to ZF‐ZR transition zone in the pseudotime analysis. Additionally, we observed an expression pattern similar to the previously described “DLK1‐expressing cell clusters”,^52^ together with some positive spots in the capsule as has been previously demonstrated in mice^73^ but not yet in humans.

Recently, it was proposed that adrenal renewal is sexually dimorphic.^43^, ^74^ In our NAG cohort, we were not able to find any significant transcriptomic differences between the two sexes, potentially due to the small sample size (tissue from two females versus four males) and the limited number (few hundred) of detected expressed genes per nucleus. Similarly, age‐specific differences were not observed, as all samples of this cohort belonged to adult subjects (ranging from 49 to 81 years of age).

Our single‐nuclei cell atlas of healthy adult human adrenal glands can be used to build up a reference to investigate adrenal‐related disease. Here, we interrogated benign adrenocortical tumours (adenomas), which were further classified according to glucocorticoid secretion and mutational status. Using this approach, high intra‐tumoral heterogeneity was observed, confirming for the first time at single‐nuclei level previous morphological and genetic findings in bulk tumours.^75^, ^76^

The comparative analysis of the transcriptional profiles of NAGs and ACAs revealed the overexpression of specific genes in all adenomas compared to normal adrenals, including *IGF2R*, *GAS2* and *SLBP*. The IGF pathway is one of the most dysregulated systems in adrenocortical tumors^53^ and *IGF2R* was found to be overexpressed in a subset of adrenocortical carcinomas.^77^ However, this is the first evidence of its overexpression in benign adrenal tumours. *GAS2* plays a major role in the regulation of microfilament and cell shape changes mostly during apoptosis.^78^ Moreover, it is involved in the Wnt‐pathway and is overexpressed in solid tumours such as colon cancer.^79^, ^80^ Like *GAS2*, *SLBP* expression is also dysregulated in colon cancer^81^ and is involved in histone stability.^82^ However, the function of both *GAS2* and *SLBP* in adrenocortical tumours remains to be further investigated.

More interestingly, we found the presence of six adenoma‐specific cell populations, including an adenoma microenvironment (AME) cluster, characterized by a lipid‐enriched milieu, similar to the tumor microenvironment described in other solid tumors.^83^ This lipid‐enriched microenvironment may play a key role in aberrant cell growth and cancer progression, as well as in immune escape and treatment‐resistance.^83^ Within the AME, we detected a high expression of *RARRES2*, a key modulator of tumour growth and inflammation, in line with a previous study reporting a higher *RARRES2* expression in benign compared to malignant adrenocortical tumours.^84^ Furthermore, we found a high expression of *IGF1R*, which plays an important role in adrenocortical tumorigenesis.^53^ Interestingly, overexpression of *IGF1R* within the tumour microenvironment was reported to promote tumour initiation and progression in lung cancer and medulloblastoma.^85^, ^86^

The “cholesterol‐ and steroid‐enriched metabolism” cluster, which showed a high expression of cholesterol pathway genes, was, as expected, largely represented in CPAs.^87^ Whereas, in the single CPA with *GNAS* mutation we observed a high prevalence of AC3, characterised by an overexpression of *PDE8A*. These results confirm previous reports that highlighted the potential role of the phosphodiesterase 8 family members in the pathogenesis of Cushing's syndrome.^14^, ^88^ On the contrary, the AC2 cluster was significantly abundant in two out of five EIA samples, where a high enrichment of post‐transcriptional modifications (including SUMOylation and deubiquitination) was detected.

High morphological heterogeneity was particularly observed in *CTNNB1*‐mutated adenomas, as recently reported in hepatocellular carcinomas harbouring mutations in the β‐catenin gene.^89^, ^90^ Based on the transcriptome profile, we observed three types of *CTNNB1*‐mutated ACA: one with a considerable abundance of AC1 (including one EIA and one CPA); one CPA with an abundance of AC4; and three EIAs without preference for a specific cell population. Of note, in the two samples with an abundance of AC1, we have observed a high expression of *AFF3*, *ISM1* and *LEF1*, all known *CTNNB1* target genes in both benign and malignant adrenocortical tumours.^55^, ^56^, ^57^ Particularly, it has been demonstrated that *AFF3* mediates the oncogenic effects of β‐catenin in ACC cell line NCI‐H295R by acting on transcription and RNA splicing.^56^ Similarly, in the AC4 abundant CPA, a high expression of *MYBL2* was found. It has been shown that *MYBL2* is a Wnt/β‐catenin target gene in different types of cancer‐xenograft models.^91^ Furthermore, *MYBL2* regulates cell proliferation, survival and differentiation and its overexpression is associated with poor outcomes in different cancer types,^92^ including ACC.^93^ These findings might support the previously reported hypothesis of a potential adenoma‐carcinoma sequence at least in a subgroup of *CTNNB1*‐mutated adrenocortical tumors.^14^

Another notable finding emerging from the transcriptional profiles of adenomas was the enrichment of elements associated with spliceosome, suggesting a potential role of the splicing process in adrenocortical tumorigenesis, as demonstrated in other benign and malignant tumours.^94^, ^95^, ^96^

Interestingly, a different distribution of immune clusters emerged among tissue entities. Both lymphoid and myeloid clusters were better represented in normal adrenals than in adenomas and myeloid cells showed a tendency to decrease in CPA compared to EIA and NAG. This finding is in line with the known interplay between glucocorticoids and tumour‐immune infiltration observed in malignant adrenocortical tumours,^97^ suggesting the inhibition of tumour‐infiltrating immune cells also in CPAs. In addition, we demonstrated for the first time a high prevalence of M2‐like macrophages within the myeloid cluster in normal adrenal. M2‐like macrophages have anti‐inflammatory properties and promote cell homeostasis, confirming the potential trophic function of adrenal macrophages.^98^ This shifted macrophage polarisation towards an M2‐like phenotype and, in particular, towards the M2c subgroup characterised by a high expression of the scavenger receptors *MRC1* and *CD163*,^99^ could be related to the autocrine action of glucocorticoids secreted by the adrenal gland. To note, in adenomas, the mutational status significantly affected the myeloid cluster, which was highly enriched in *CTNNB1*‐mutated adenomas. As observed in NAG, adenoma‐associated macrophages presented a prominent M2‐like polarization, corroborating previous studies reporting that Wnt/β‐catenin signalling promotes M2‐like macrophage in tumors^100^ and that the M2 signature is highly prevalent in *CTNNB1*‐mutated samples.^101^

In conclusion, using snRNA‐seq and spatial transcriptomic analysis of adult human adrenal glands we could identify complex mechanisms of the normal adrenal gland homeostasis, as well as molecular processes that could be involved in early adrenocortical tumorigenesis and autonomous cortisol secretion in adenomas.

## 4 MATERIALS AND METHODS

### 4.1 Tissue sample selection

For snRNA‐seq, nuclei were isolated from six snap‐frozen samples collected from NAGs deriving from the tissue surrounding EIA or from adrenalectomies performed during surgery for renal cell carcinoma (Figure S1). Demographic data and details of detected cellular transcriptomes are summarized in Table 1. Additionally, single nuclei were isolated from 12 fresh‐frozen ACA samples, including 7 CPAs and 5 EIAs. Among these samples, four tissues were included in two previous studies on whole‐exome sequencing^16^ or bulk RNA‐sequencing.^14^ Clinical characteristics, hormonal secretion, and detected cellular transcriptomes are summarized in Table 2 and Table S3.

Validation of the results found for the NAGs was done by immunohistochemistry (IHC). For IHC, 16 formalin‐fixed paraffin‐embedded (FFPE) tissue sections of NAGs were analysed. This set included three NAGs that were also part of the snRNA‐seq analysis and one additional sample used for the Visium analysis (Table S4). Among these 16 cases, we used the remaining FFPE‐slides for RNAscope in situ hybridization (*n* = 14) or immunofluorescence (*n* = 5). Tissue samples were selected from the Würzburg Adrenal Biomaterial Archive, part of the BMBF‐funded Interdisciplinary Bank of Biomaterials and Data Würzburg (IBDW)^102^ (details in Supporting Information). Moreover, an additional three FFPE slides were added for immunofluorescence analysis (Table S4).

The study was approved by the ethics committee of the University of Würzburg (Nos. 93/02 and 88/11) and written informed consent was obtained from all subjects.

### 4.2 Sequencing of driver mutations in adenoma tissues

Known driver hot‐spot mutations in *CTNNB1* (exon 3), *PRKACA* (p.Leu206) and *GNAS* (p.Arg201 and p.Gln227)^14^, ^16^ were evaluated by Sanger sequencing in bulk tumour tissues (*n* = 8). Four samples were analysed by Whole Exome Sequencing or RNA‐seq as part of previous studies from our group.^14^, ^16^ DNA was isolated from snap‐frozen ACA tissues with the Maxwell 16 Tissue DNA Purification Kit (#AS1030; Promega) according to the manufacturer's instructions. The mutations were genotyped by polymerase chain reaction, as previously reported^103^ (details in Supporting Information).

### 4.3 Clinical data collection

For ACAs, the endocrine workup was conducted according to the adrenal incidentalomas guidelines issued by the European Society of Endocrinology/European Network for the Study of Adrenal Tumors^10^ and the Endocrine Society guidelines for the diagnosis of Cushing's syndrome.^104^ Hormone levels were measured using commercially available analytical procedures, as previously reported^105^ (details in Supporting Information).

### 4.4 snRNA sequencing

#### 4.4.1 Single‐nuclei isolation

We applied a previously published protocol for nuclei isolation from snap‐frozen tissue^106^ (Figure S1 and Supporting Information). The nuclei integrity and purity were checked under a microscope and the yield was quantified using a Neubauer chamber.

#### 4.4.2 snRNA sequencing

We used the inDrop method, which relies on poly A tails to capture transcripts, consisting of three major steps: Silanization, Reverse Transcription and Library preparation (1CellBio) following the manufacturer's protocols (Supporting Information).

After the library preparation step, the six NAG, two endocrine‐inactive adenomas (EIA‐3 and EIA‐4) and two cortisol‐producing adenomas (CPA‐1 and CPA‐2) libraries were pooled together and sequenced using an Illumina HiSeq 2000 instrument with 60 bases for read (R1), 6 for the Illumina index and 50 for the read 2 (R2). The remaining eight adrenocortical adenoma libraries were pooled together and sequenced on two NovaSeq S1 flow cells at 2 × 100 configurations. The six NAGs were integrated using the standard Seurat (version 3.0) integration pipeline^107^ (freely available software). The resulting data is shown in Figure S1C. Cells from the two subtypes showed a similar clustering pattern, suggesting that the data were well integrated. Of note, data derived from either NAG‐EIA or NAG‐RCC patients showed a correlation with a high Pearson correlation score of 0.99 (Figure S1D), indicating that NAGs could be merged in one single group.

#### 4.4.3 Generation of count matrices and core processing

The resulting base calls (BCL) files were converted to FASTQ files using the bcl2fastq tool (Illumina). These raw sequencing reads were processed with the zUMI^108^ (version 2.5 – default parameters) pipeline. The count matrices from reads spanning both introns and exons (inex) were used for subsequent analysis. Prior to pre‐processing, the genes/features were converted from Ensembl (ENSG) IDs to gene symbols using a custom script. The same cut‐off was used to filter nuclei and genes likewise from individual samples (min.features = 200 and min.cells = 3, *CreateSeuratObject*). The fraction of reads mapping to the mitochondrial chromosome (MT‐) was calculated (*PercentageFeatureSet*).

#### 4.4.4 Data normalization and integration using Seurat

The filtered data was log‐normalized (*NormalizeData*) and 2000 highly variable genes (HVG) were selected. The six samples were then integrated (1. *FindIntegrationAnchors, 2. IntegratedData*).^109^ The integrated data was then scaled (*ScaleData)* and the effect of mitochondrial genes was regressed.

#### 4.4.5 Dimensionality reduction and clustering

Thirty principal components (PCs) were computed (*RunPCA*) and the first 15 were used to generate the UMAP reduction (*RunUMAP*). Subsequently, the k‐nearest neighbours (knn) were determined (*FindNeighbors*) and clustering was performed (*resolution = 0.01, FindClusters*). The resulting clusters were annotated using known marker genes and differential gene expression (DGE—*FindAllMarkers*) analysis.

#### 4.4.6 Module scoring

A list of top DEGs and prior knowledge was curated for each cluster, and combined gene expression scores were calculated (*AddModuleScore*). The Seurat object was further filtered using these scores, with the following thresholds: ZG > 0.3, ZF > 0.8, ZR > 0.5, MC > 0, LC > 0, FC > 0, VEC > 0, AM > 0 and AMP > 0. This resulted in a count matrix of 5154 nuclei that was then integrated with the ACA dataset. A Shiny implementation (R) was set up to easily navigate within this data.

#### 4.4.7 Gene enrichment analysis

We used pathfindR^110^ to perform the gene enrichment analysis using the *FindAllMarkers* output table from Seurat. The analysis was performed using the function *run_pathfindR* for both NAG and ACA samples. For the NAGs, KEGG pathways were used as a reference. To better cover tumour heterogeneity in ACAs, three different databases (KEGG, Reactome and GO‐All) were used as references.

### 4.5 Visium (10x Genomics) spatial gene expression assay

Frozen adrenal gland samples (NAGs) were embedded in OCT (TissueTek) and cryo‐sectioned (Thermo Scientific, CryoStar NX50) with a 10 µm thickness. The sections were then placed on tissue optimization and gene expression slides. 20 min was determined as the optimal permeabilization temperature. Bright‐field H&E images were taken with a 10X objective (Leica DMi8 – A). After recovery of cDNA from the slide, the libraries were prepared following the gene expression user guide (10x Genomics—CG000239 Rev A). Subsequent to pooling, libraries were loaded at 200 pM and sequenced on a HiSeq‐4000 (Illumina).

#### 4.5.1 Generation of Visium count matrices

The base call (BCL) files generated from the raw sequencing data were converted to FASTQ reads using the bcl2fastq tool (Illumina). The FASTQ reads were mapped to the human reference dataset GRCh38 (build 2020‐A; *refdata‐gex‐GRCh38‐2020‐A*) using the Space Ranger v1.1.0 *count* pipeline. On average the two slides captured every 1269 spots under the tissue, and both yielded 1973 median genes and 225,346 mean reads per spot.

#### 4.5.2 Seurat processing of Visium count data

The two Visium Seurat objects were created using default parameters (*Load10X_Spatial*) and were subsequently normalised (*SCTransform*). Thirty PCs were computed and the first 20 were used to generate the UMAP reduction. Knn of each spot was then determined (*FindNeighbors*) and clustering was performed (*resolution = 0.2*, *FindClusters*), yielding 6 spatial clusters.

#### 4.5.3 Label transfer from snRNA‐seq data onto Visium

Label transferring was performed according to the label transfer workflow in Seurat. Anchors or cell‐spot pairwise comparisons were computed (*FindTransferAnchors*), and subsequently, a prediction assay was generated (*TransferData*), containing the prediction scores for each cell type within each spot. Spatially variable feature lists were generated and included in both objects (*assay = “SCT”*, *selection.method = “markvariogram”* and *FindSpatiallyVariableFeatures)*.

### 4.6 Trajectory analysis

Both Visium objects were integrated with the SCTransform integration framework using default parameters. The Louvain clustering was performed (*resolution = 0.2*) and those were annotated with prior knowledge from the label transfer analysis. The SCT adjusted count matrix (“counts” slot in SCT assay) was extracted from the integrated object, and a “CellDataSet” object was generated (*new_cell_data_set*) within Monocle 3.^48^ Subsequently, the cds object was normalized (*preprocess_cds*) and Seurat computed UMAP coordinates was added into reducedDims slot. The trajectory was then fit (*close_loop = F, use_partition = F*, *learn_graph*) and pseudotime was computed (*order_cells*) by setting the root to *RSPO3+* cells. After getting the genes that vary as a function of pseudotime (*neighbor_graph = “principal_graph”*, graph_test), they were grouped into modules (*resolution = 0.02, find_gene_modules*). Genes from top‐scoring modules (0 for Capsule, 1 for ZG and 2 for ZF_ZR) were combined to construct the top gene list that differentially changes across pseudotime.

### 4.7 ACA‐NAG data integration

#### 4.7.1 Count matrix merging and normalization

Count matrices from 12 ACA (37 037 nuclei: 7 CPA and 5 EIA) and highest scoring 5154 NAG nuclei (See Module Score section above) were merged. The Seurat object was then generated (*min.features = 200*, *min.cells = 3* and *CreateSeuratObject*), cell cycle (S.Score, G2M.Score) and mitochondrial genes were scored (*PercentageFeatureSet)* and regressed out during the normalisation step with SCTransform.^111^

#### 4.7.2 Integration using harmony

Post normalisation, sample integration was performed within the Harmony framework (1. *RunPCA*, *2. RunHarmony with 40 PCs*).^112^ Knns were computed (*FindNeighbors*) on the UMAP dimension and clustering was done (*resolution = 0.2*, *FindClusters*). Finally, a UMAP reduction was performed on the Harmony dimensions (*RunUMAP*). Clusters with less than 5 differentially expressed features were removed.

#### 4.7.3 Analysis of DEG among the ACA clusters

A logistic regression test was used within the FindMarkers framework implemented in Seurat for computing DEGs across subtypes (NAG, EIA and CPA) and clusters. The results were visualised as volcano plots (EnhancedVolcano v1.6.0) and heatmaps (ComplexHeatmap v2.4.3)

#### 4.7.4 Cluster distribution among tissue samples

The distribution of the lymphoid and myeloid clusters among the samples was evaluated across NAG, EIA and CPA. Moreover, lymphoid and myeloid cluster distribution was evaluated within the adenomas considering the mutational status (no known driver vs. *CTNNB1*‐mutation vs. *PRKACA/GNAS*‐mutation). The distribution of the adenoma‐specific clusters was evaluated within the adenoma samples considering both the secretion types (EIA vs. CPA) as well as the mutational status (no known driver vs. *CTNNB1*‐mutation vs. *PRKACA/GNAS*‐mutation). To this aim, the percentage of the number of cells within the cluster per sample was considered. Data were analyzed using the non‐parametric Mann‐Whitney U test and the Kruskal‐Wallis test, followed by Dunn's multiple comparison test, as appropriate. A *p*‐value < 0.05 was considered statistically significant. The statistical analysis was performed with GraphPad Prism version 9 (GraphPad Software).

### 4.8 Immunohistochemistry

IHC was used to validate the expression and spatial context of the proteins of selected genes from the transcriptome analysis. This assay was performed with FFPE material from 16 NAGs with available slides (Table S4), as previously described.^113^ Primary antibodies are summarised in Table S5.

Double immunostaining was applied to validate the two newly identified cell populations. The combination of NR2F2/ID1 and CHGA/SYT1 was used to test FFPE consecutive sections from nine NAGs. Details of the IHC assay including image acquisition are reported in the Supporting Information.

### 4.9 RNAscope in situ hybridization

To validate ID1^+^ cells within the vascular endothelial cell cluster, we performed RNAscope in situ hybridization (ISH) in NAG tissues. This ISH assay was performed in the FFPE tissue section from 14 NAGs with available FFPE material (Table S4) using the RNAscope 2.5 HD Assay for chromogenic dyes (Cat No. 322300, Advanced Cell Diagnostics, ACD a Bio‐Techne brand) following the manufacturer's instructions. Details on the method and image acquisition and analysis are reported in the Supporting Information.

### 4.10 Immunofluorescence

Immunofluorescence for CYP17A1/SYT1 and NR2F2/Ki67 double staining were performed in 8 FFPE‐slides of NAG (Tables S4 and S5). To avoid autofluorescence, the Vector TrueVIEW Autofluorescence Quenching Kit with DAPI (SP‐8500‐15, Vector Laboratories) was used according to manufacturer instructions. Further details are available in Supporting Information.

## AUTHOR CONTRIBUTIONS

Cristina L. Ronchi, Martin Fassnacht and Somesh Sai designed and coordinated the study and provided funding. Barbara Altieri, Laura‐Sophie Landwehr and Cristina L. Ronchi collected the tissue samples. A. Kerim Secener, Panagiota Arampatzi, Sarah N. Vitcetz and Caroline Braeuning prepared the samples for the sequencing and generated sequencing data. A. Kerim Secener, Somesh Sai and Cornelius Fischer performed bioinformatics analysis. A. Kerim Secener performed Visium experiments. Stefan Kircher provided the analysed tissues. Barbara Altieri, Silviu Sbiera and Stefan Kircher performed immunohistochemistry. Sabine Herterich evaluated the mutational status of the included adenomas. Barbara Altieri and A. Kerim Secener performed statistical analysis and generated figures. Data interrogation and interpretation were carried out by Barbara Altieri, A. Kerim Secener, Somesh Sai, Cornelius Fischer, Silviu Sbiera, Martin Fassnacht, Cristina L. Ronchi, Silviu Sbiera, Barbara Altieri, A. Kerim Secener, Cristina L. Ronchi and Sascha Sauer wrote the manuscript and Silviu Sbiera, Laura‐Sophie Landwehr and Martin Fassnacht provided critical feedback. All other authors read and approved the manuscript.

## CONFLICT OF INTEREST STATEMENT

The authors declare no conflict of interest.

## FUNDING INFORMATION

This work has been supported by the Deutsche Forschungsgemeinschaft (DFG) (project FA‐466/8‐1, RO‐5435/3‐1 and 405560224 to M.F., C.L.R. and S.S.) and within the CRC/Transregio (project number: 314061271‐TRR205), and the Deutsche Krebshilfe (70113526 to M.F. and C.L.R.). This work has been carried out with the help of the Interdisciplinary Bank of Biomaterials and Data of the University Hospital of Würzburg and the Julius Maximilian University of Würzburg (IBDW), supported by the Federal Ministry for Education and Research (Grant number FKZ: 01EY1102).

## ETHICS STATEMENT

The study was approved by the ethics committee of the University of Würzburg (No. 93/02 and 88/11) and written informed consent was obtained from all subjects.

## Supporting information

Supporting Information

Supporting Information

## Data Availability

Sequencing data was submitted to the ArrayExpress database of the European Bioinformatics Institute (EBI); accession number: E‐MTAB‐12129. Visium data can be retrieved from here: https://doi.org/10.5281/zenodo.5289292.
