# Supplementary material for: Single‐nucleus and spatial transcriptome reveal adrenal homeostasis in normal and tumoural adrenal glands
Source: Clin Transl Med. 2024 Aug 21;14(8):e1798. doi: 10.1002/ctm2.1798 (PMC11338279; doi:10.1002/ctm2.1798)
Supplement: Supplementary file 1 — Supporting Information [file CTM2-14-e1798-s002.docx]

Supplemental Materials for

**Single-Nucleus and Spatial Transcriptome Reveal Adrenal Homeostasis in Normal and Tumoral Adrenal Gland.**

Barbara Altieri, A. Kerim Secener, Somesh Sai, Cornelius Fischer, Silviu Sbiera, Panagiota Arampatzi, Stefan Kircher, Sabine Herterich, Laura-Sophie Landwehr, Sarah N. Vitcetz, Caroline Braeuning, Martin Fassnacht^*^, Cristina L. Ronchi^*^, Sascha Sauer^*^

*Corresponding author. Email: fassnacht_M@ukw.de (M.F.); C.L.Ronchi@bham.ac.uk (C.L.R); sascha_sauer_2020@gmx.de (S.S)

**Supplemental Materials**

**Supplemental Methods**

***Tissue sample collection:*** Subsequent to surgery, patient materials were collected and immediately stored at -80°C after flash freezing. The dissection of the adrenal tissues as well as of the adrenocortical adenomas was made by expert pathologists (1). The tissues with an average size of about 5x5x5 mm were ground gently using a mortar and a pestle in liquid nitrogen until obtaining a granular powder. Hereafter, half of the ground tissue was conserved at -80°C and the other half was used for downstream processing.

The aforementioned patient materials were selected from Würzburg Adrenal Biomaterial Archive, part of BMBF-funded Interdisciplinary Bank of Biomaterials and Data Würzburg (IBDW) applying the highest standards for biobanking (2).

***Clinical data collection:*** Hormone levels were measured using commercially available analytical procedures: serum cortisol and adrenocorticotropic hormone (ACTH) were analyzed by Immulite 2000 Xpi from Siemens; late-night salivary cortisol (LNSC) was measured by a manual luminescence immunoassay from IBL; 24-h urinary free cortisol (UFC) was analyzed by a manual radioimmunoassay from Immuntech, as previously reported (3).

The following screening tests for the diagnosis of cortisol-producing adenomas were applied as follows: 1 mg dexamethasone suppression test (DST) with a cutoff value of 50 nmol/l, LNSC (assay-specific reference normal range 0-4.1 nmol/L) and/or 24-h UFC (assay-specific reference normal range 22-193 nmol/d) (4).

***Single-nuclei isolation:*** Ground snap-frozen adrenal tissue was homogenized in a Triton X-100 based lysis buffer (10% Triton X-100, Superasin 20 U µL^.1^, RNaseIn U µL^-1^, Nuclei Isolation Medium: 1 mM DTT, 50x Protease Inhibitor, 1.5M Sucrose, 1M KCl, 1M MgCl2, 1M Tris buffer pH 8.0). The homogenized nuclei were stained with DAPI (4’,6-diamino-2-phenylindole) and sorted by FACS (BD FACSAria^TM^ Fusion cell sorter, BD Genomics, USA) into RNAse inhibitor containing tubes (Supplemental Figure 1).

***Single-nuclei library preparation:***

First, chips were silanized, primed with the inDrop hydrogel beads, droplet making oil, RT/lysis mix and the nuclei. After completing the run protocol, cell encapsulations were collected and exposed to UV light to release photocleavable primers from hydrogel beads. Subsequently, reverse transcription was performed, and the beads filtered out. From there on, the library preparation step followed the CEL-Seq 2 protocol (5): the RT product was digested by ExoI and HinFI and purified using AMpure XP beads (Beckman Coulter^TM^, USA). Then, the second strand cDNA synthesis and *in vitro* transcription (IVT) steps were carried out. The resulting RNA was subjected to fragmentation and then reverse-transcribed using random hexamers. The cDNA was ultimately purified using AMpure XP beads and quantified by qPCR assay (6) (Roche Light Cycler 480 Instrument II, Switzerland).

***Immunohistochemistry***

Briefly, consecutive formalin-fixed paraffin-embedded (FFPE) serial sections of 16 NAGs were deparaffinized and rehydrated in descending graded series of ethanol. High-temperature antigen retrieval was achieved in 10 mM citric acid monohydrate buffer (pH 6.5) in a pressure cooker. Blocking of unspecific binding was performed with 20% human AB serum in PBS for 1 h at room temperature. Primary antibodies (summarized in Supplemental Table 5) were incubated 1 hour at room temperature. As negative control, tissue sections were incubated with N-Universal Negative Control Anti-Rabbit or Anti-Mouse (IS600 and IS750, respectively, Dako, Glostrup, Denmark), depending on the primary antibody used. Signal amplification was achieved by HiDef Detection HRP Polymer System (954D-50, Medac Diagnostika, Germany) followed by 10 min development with DAB substrate kit (957D-30, Cell Marque, USA). Nuclei counterstaining was obtained using with Mayer’s haematoxylin for 2 min (T865.1, Carl Roth, Germany).

Double immunostaining was used to validate the markers of the newly found cell populations. The combination of NR2F2/ID1 and CHGA/SYT1 was used. FFPE consecutive section from 9 NAGs were evaluated. Blocking of unspecific binding was performed using 20% human AB serum in TBS solution. Slides were incubated with NR2F2 or CHGA antibodies and visualized in fuchsin-red by alkaline phosphatase MACH 3 Mouse AP-Polymer Detection (M3M532, Biocare Medical, CA, USA) and Vulcan Fast Red Chromogen Kit 2 (FR805H, Biocare Medical, CA, USA). A following incubation with secondary primary antibodies ID1 or SYT1 was performed. After signal amplification and detection with HiDef Detection HRP Polymer System, ID1 or SYT1 were stained in blue-green by Vina Green Chromogen (BRR807AH, Biocare Medical, CA, USA). Nuclei counterstaining was obtained using with Mayer’s haematoxylin for 1 min.

All images were acquired by Leica Aperio Versa Brightfield scanning microscope (Leica, Germany) up to 40x magnification (HPF) using same light intensity parameters to avoid biased information. Evaluation of the nuclear staining of ID1 and NR2F2, as well as staining SYT1, was performed by automated image analysis (Aperio ImageScope software). The non-parametric Mann-Whitney U test was used for evaluating the differences of SYT1 staining among cells of the cortex, medulla and SYT1^+^ niches (GraphPad Prism version 9, GraphPad Software, San Diego, CA, USA). The evaluation of positive areas for SYT1 were evaluated with ImageJ software using three pictures at 20x magnification of representative areas of each slide. Only strong stained cells were considered positive for SYT1 (stained background was excluded from the analysis).

***RNAscope in situ hybridization***

Consecutive 2 µm thickness FFPE tissue sections from 14 NAGs were used for the evaluation of *ID1* by RNA *in situ* hybridization. Briefly, FFPE slides were deparaffinized in xylene and washed with 100% ethanol. Endogenous enzyme blocking was achieved in hydrogen peroxide solution (322335, Advanced Cell Diagnostics, ACD) at room temperature for 10 min and followed by permeabilization in a pressure cooker with target retrieval reagent (322000, ACD). Protein digestion was performed using the Protease Plus (ref. 322331, ACD) for 20 min at 40°C in a HybEZ Oven (from ACD). Probes for ID1 (Hs-ID1, 414351) were hybridized at 40°C for 2h. The probe for Cyclophilin B (PPIB, 313901) was used as positive control, whereas the probe for the dihydrodipicolinate reductase (DapB, 310043), a bacterial gene, was used as negative control. Following washing steps were performed using the wash buffer (31009, ACD). Signal amplification was achieved by six different steps and amplifier solutions: 40°C for 40 min with Amplifier 1 (322311, ACD) and 3 (22313, ACD) alternating with Amplifier 2 (322312, ACD) and 4 (322314, ACD) at 40°C for 20 min, and followed by Amplifier 5 (322315, ACD) for 50 min and 6 (322316, ACD) for 20 min both at room temperature. Chromogenic detection was obtained by a mixed solution of DAB-A (320052, ACD) and DAB-B (320053, ACD) for 10 minutes at room temperature. Nuclei were counterstained with Meyer’s Hematoxylin (T865.1, Carl Roth, Germany) and the slides were mounted using Entellan (Merck, Germany).

For the evaluation of the RNAscope, three pictures at 40x HPF of representative areas of each slide were taken with the Leica Aperio Versa brightfield scanning microscope (Leica, Germany). The scoring of mRNA expression was assessed with automated image analysis ‘RNA ISH v1’ algorithm by Aperio ImageScope software (version 12.x, Leica, Germany). The number of dot/cells were also evaluated.

***Immunofluorescence***

FFPE slices of 2 or 5 µm thickness from 8 NAGs were used for the immunofluorescence (IF) analysis (Supplemental Table 4). Deparaffinization and rehydration was performed in xylene followed by descending graded series of ethanol. Antigen retrieval was obtained in 10 mM citric acid monohydrate buffer (pH 6.5) in a pressure cooker. Permeabilization was performed with 10% goat serum (EMD Millipore Corporation, Temecula, USA) and 0.5% Triton-X (3051.3, Carl Roth) in PBS for 10 min, following by blocking of unspecific binding by 10% goat serum and 1% bovine serum albumin (BSA, Sigma) in PBS for 30 min at room temperature. Slides were then co-incubated with primary antibodies anti-CYP17A1/SYT1 and anti-NR2F2/Ki67 overnight at 4°C in a humidified chamber (Supplemental Table 5). As negative controls, tissue sections were incubated 1% BSA in PBS without antibody cocktail. Slides of adrenocortical carcinoma, breast cancer and colon cancer were used as positive controls for the NR2F2/Ki67 staining. After washing steps with PBS, tissue sections were incubated with secondary antibodies anti-mouse conjugated Alexa Fluor 488 (ab150117, Abcam, 1:300) and anti-rabbit conjugated Alexa Fluor 555 (A21428, Thermo Fisher, 1:200). To avoid autofluorescence, 150 µl of Vector TrueVIEW reagent (SP-8500-15, Vector^®^ TrueVIEW^®^ Autofluorescence Quenching Kit with DAPI, Vector Laboratories) were added to each slide and incubate for 3 min, according to manufacture instruction. After washing in PBS buffer for 5 min, mounting was perfomed by adding VECTASHIELD Vibrance Antifade Mounting Medium with DAPI (from SP-8500-15 kit) onto tissue sections.

Images were acquired within 48 hours of mounting.by Leica Aperio Versa Brightfield scanning microscope (Leica, Germany) up to 40x HPF.

**Supplemental Figures**

**Supplemental Figure 1. Sample preparation workflow**

**A.** Six normal human normal adrenal glands (3 NAGs from endocrine inactive adenoma “NAG-EIA” and 3 from renal cell carcinoma “NAG-RCC” patients) and 12 adrenocortical adenoma (ACAs: 5 from EIA and 7 from cortisol-producing adenoma (CPA) patients) samples were collected and snap-frozen. The dissection of the adrenal gland was done by an expert pathologist. Nuclei extraction was performed via dounce homogenization (7). Using FACS, nuclei were sorted into tubes via DAPI discrimination. **B.** Subsequently, the samples were processed with the inDrop**^TM^** (1CellBio) system, according to the manufacturer’s protocol. **C.** UMAP representation of the single-nuclei transcriptomes from the six normal samples (NAG-EIA in red; NAG-RCC in blue). **D.** Average gene expression correlation between NAG-EIA and NAG-RCC patients (Pearson correlation coefficient (r = 0.99).


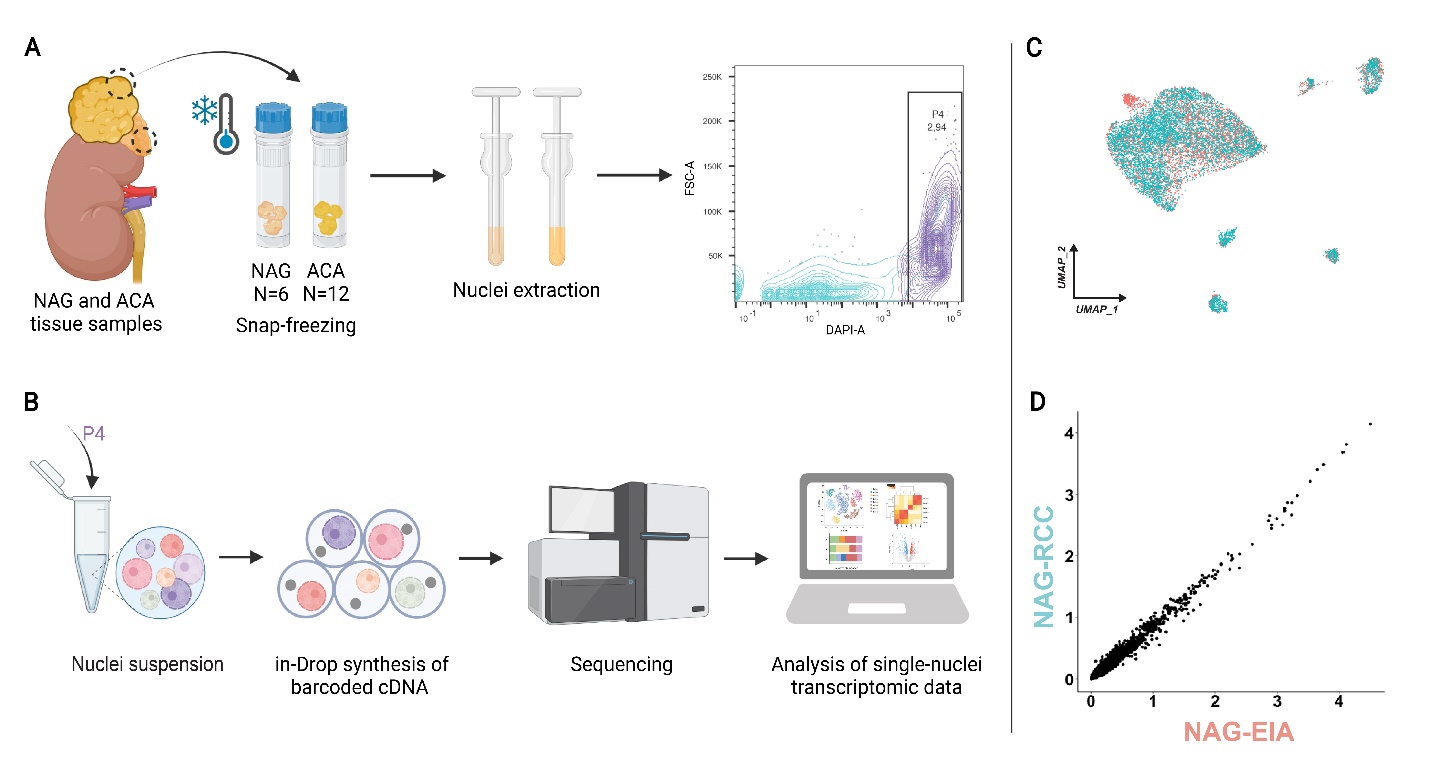


**Supplemental Figure 2. Violin plots representing cluster-specific gene expression in adult human normal adrenal glands at single-nuclei transcriptome level**

**A-C:** Subclusters of the normal adrenal cortex: zona glomerulosa, zona fasciculata, and zona reticularis. **D:** adrenal medulla. **E-I:** Satellite clusters of the normal adrenal cortex, namely, myeloid cells, lymphoid cells, fibroblasts and connective tissue, vascular endothelial cells, and cortical-neuroendocrine cells.


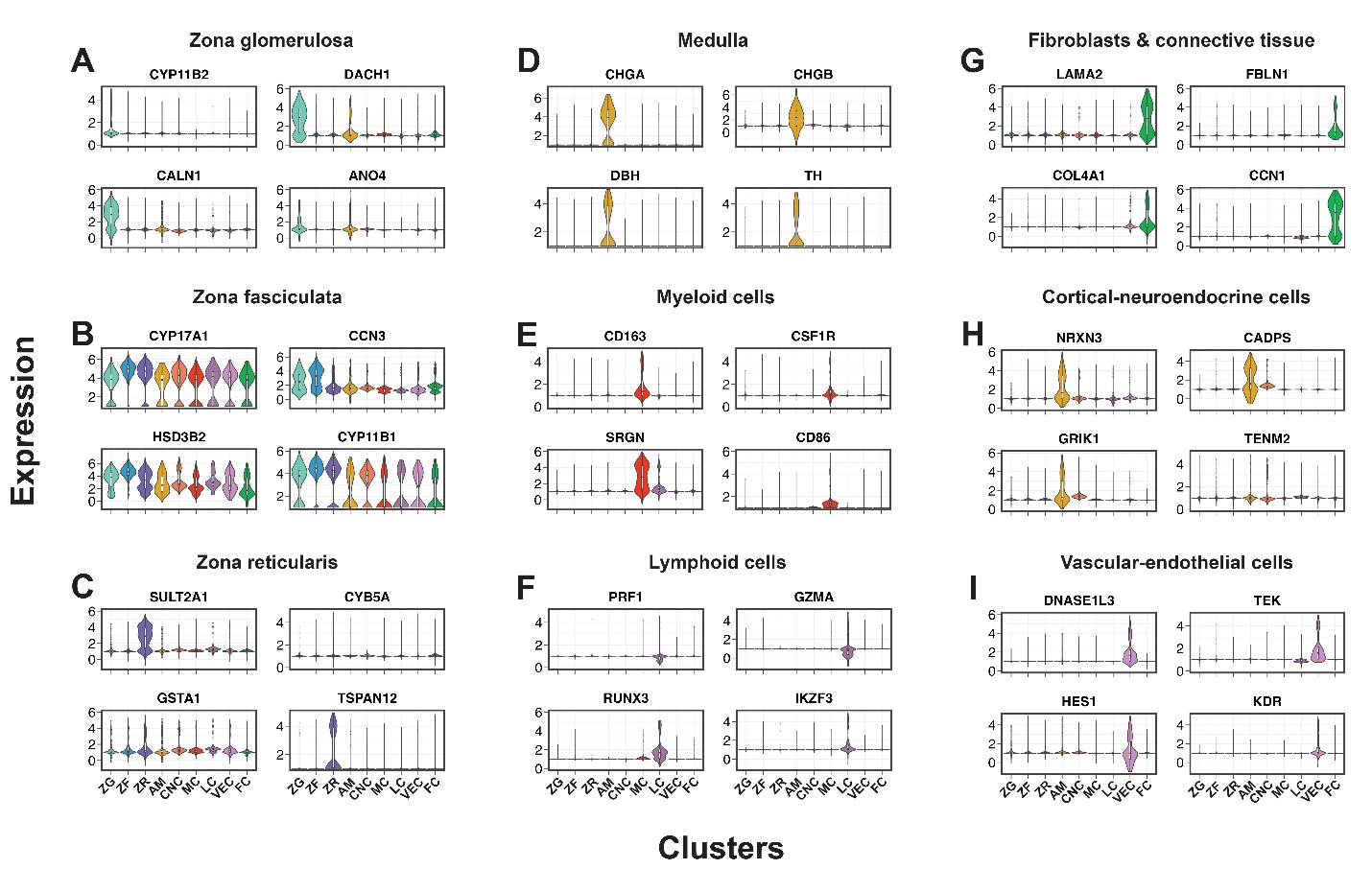


**Supplemental Figure 3. Mapping adrenal cortex zonation through correlations between single-nuclei transcriptome clusters and immunohistochemical markers**

**A.** Haematoxylin and eosin (H&E) staining of a tissue sample of normal adrenal gland, showing the three zones of the cortex (zona glomerulosa, ZG; fasciculata, ZF; reticularis, ZR) together with the capsule (C) and the adrenal medulla (M)**.** **B-D**. Kernel density estimation for selected RNA markers and their corresponding protein staining using immunohistochemistry (IHC) for each of the three adrenocortical zones: the zona glomerulosa with CYP11B2, DACH1, the zona fasciculata with CYP17A1, and the zona reticularis withCYB5A, SULT2A1. CYP11B2 staining was confirmed to be expressed in the ZG as sporadic and scattered cells underneath the adrenal capsule or as clusters of cells (previously termed as aldosterone-producing cell clusters). DACH1 was expressed in the nuclei of the ZG cells. CYP17A1 was strongly expressed in the cytoplasm of adrenocortical cells of ZF and ZR, but not in the ZG. CYB5A1 was selectively stained in the cytoplasm of cells of the ZR, whereas SULT2A1 was strongly stained in the ZR, although in the ZF a low staining was observed. **E-G.** Kernel density estimation for selected RNA markers and their corresponding protein staining for the evaluation of adrenal medulla (expressing CHGA), fibroblast and connective tissue (expressing COL1A2, MGP) and vascular-endothelial cells (expressing ENTPD1).

All images were acquired by Leica Aperio Versa brightfield scanning microscope (Leica, Germany). Scale bar = 3000 µm in pictures representing the entire adrenal gland, and 100 µm in the enlarged pictures.


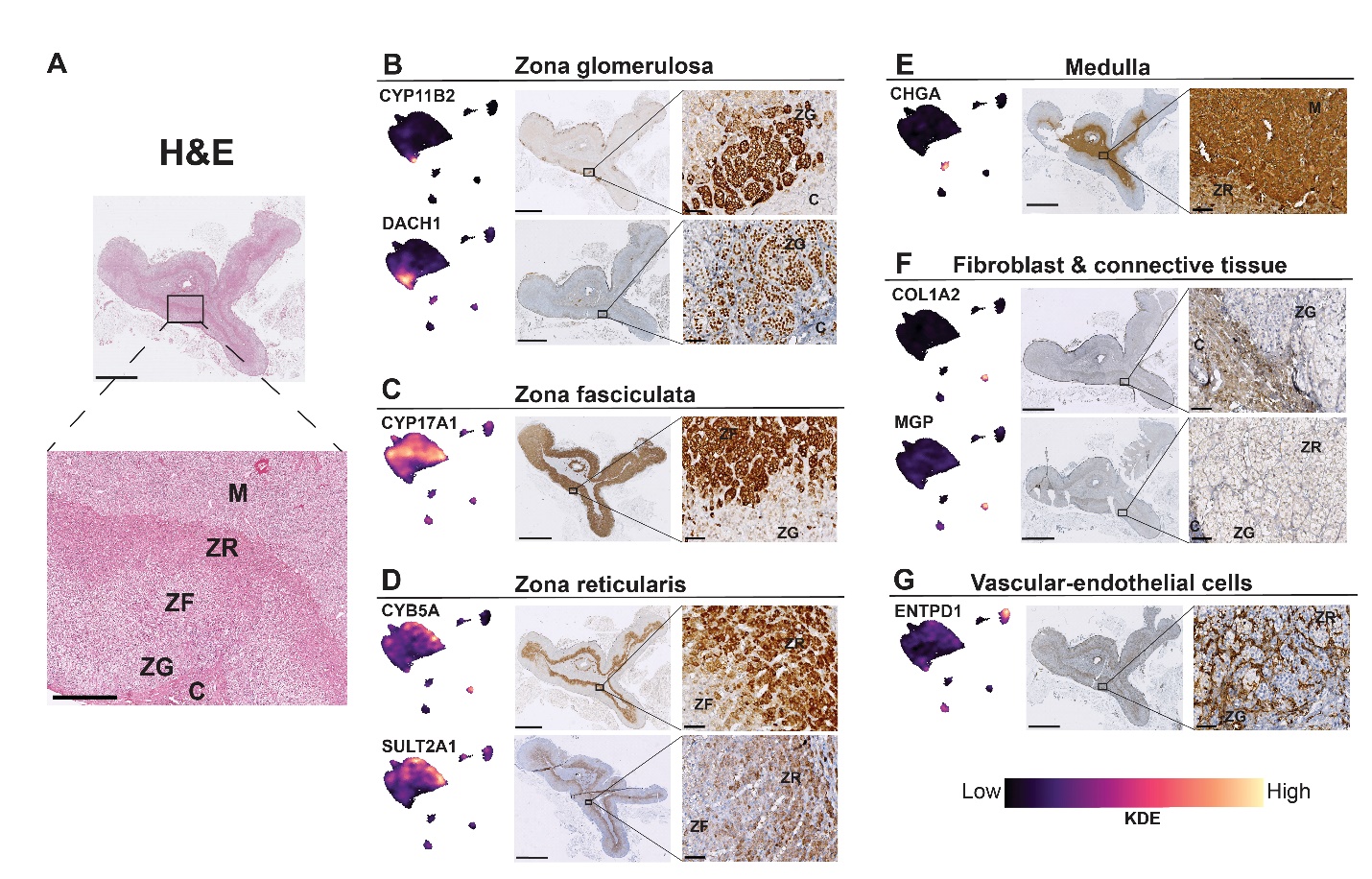


**Supplemental Figure 4. Validation of the ID1^+^ and NR2F2^+^ cells by RNAscope and immunofluorescence.**

A) RNA *in situ* hybridization was used for the validation of the ID1^+^ cells because of the lack of high specificity of most commercially available antibodies. *ID1^+^* positive cells were sporadic or formed groups of cells usually located around the adrenocortical cells of the ZG and ZF. Positive *ID1* dots are indicated with arrows. B) Sporadic endothelial cells positive for the proliferative marker Ki67 were found within the adrenal cortex. C) immunofluorescence (IF) for NR2F2 and Ki67 in one normal adrenal gland (NAG) and one adrenocortical carcinoma (ACC) used as positive control. Co-staining IF demonstrated that NR2F2^+^ cells (in green) are different from the Ki67^+^ (in red). All images were acquired by Leica Aperio Versa brightfield scanning microscope (Leica, Germany). Scale bar: 100 µm.


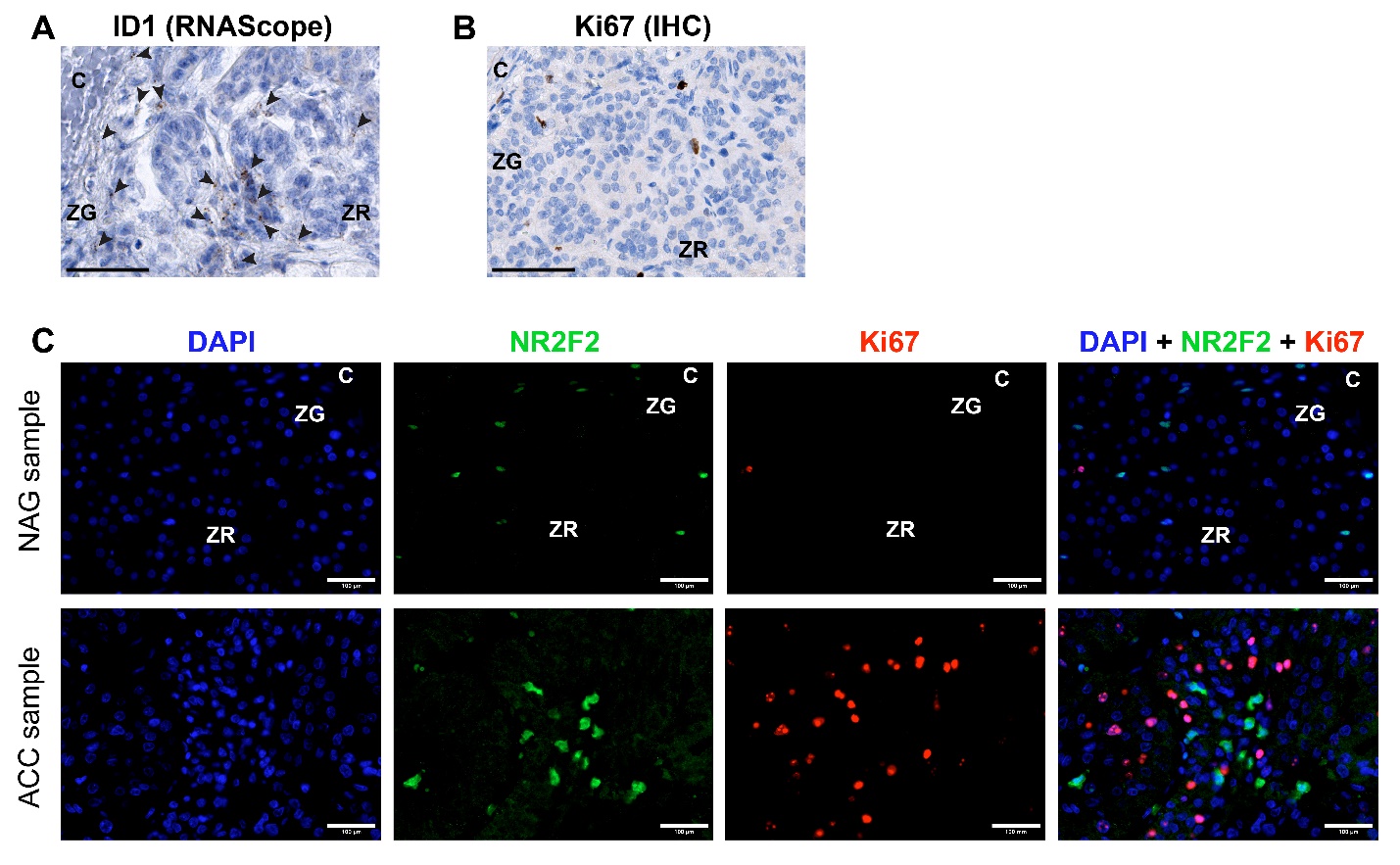


**Supplemental Figure 5. Expression of sympathoadrenal and steroidogenesis genes in the three cortex clusters as well as in the cortical-neuroendocrine cells.**

Normalized expression of *NR5A1*, *STAR, CYP11A1, CYP17A1*, *HSD3B2*, and *CYP11B1* (**A**), and normalized expression of *SYT1* and *NRG1* (**B**) in the zona glomerulosa (ZG), zona fasciculata (ZF), zona reticularis (ZR) and cortical-neuroendocrine cells (CNC).

**
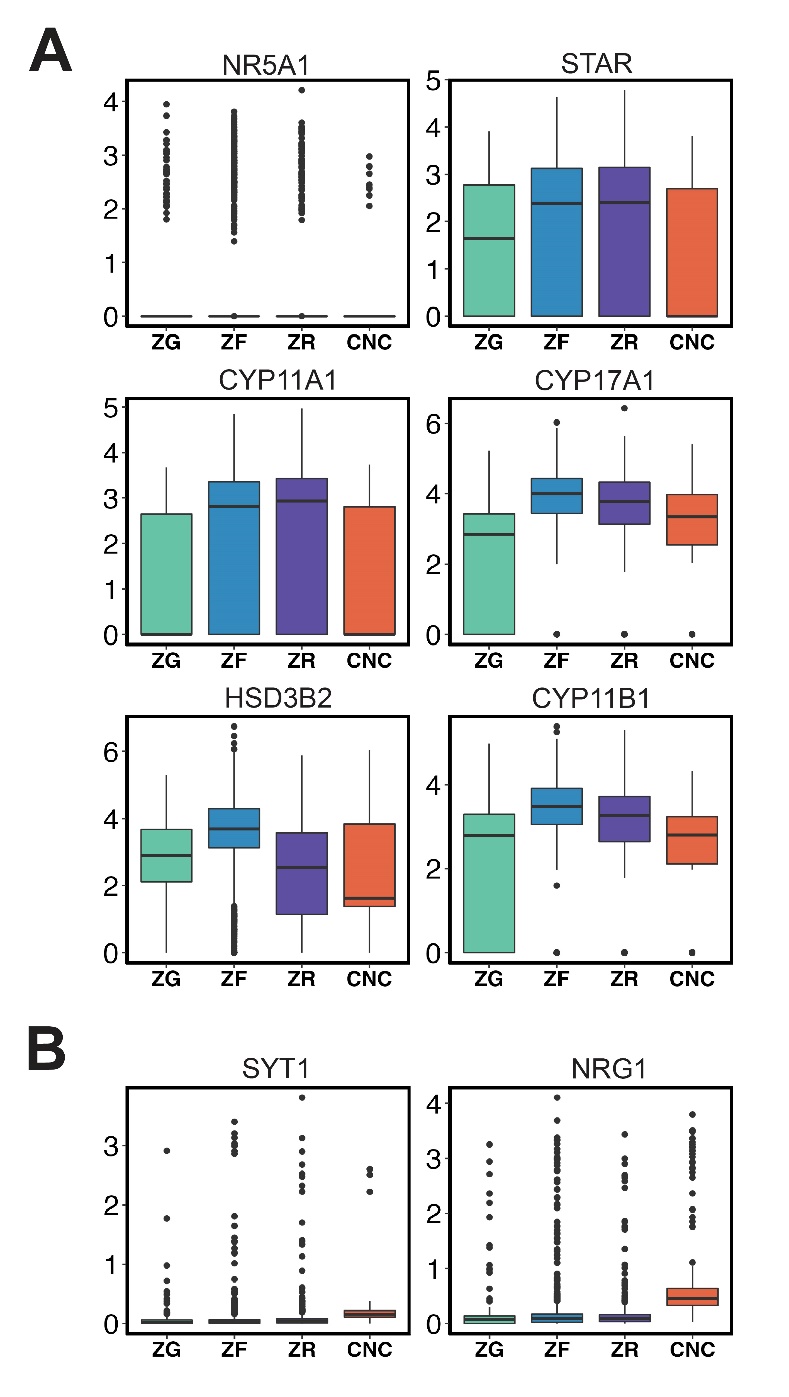
**

**Supplemental Figure 6. Immunohistochemical localization of SYT1^+^ cortical-neuroendocrine cells.**

Immunohistochemistry (IHC) showed the presence highly positive sporadic SYT1^+^ cells found in within the adrenal cortex (A-B). Picture B showed the contrast between highly SYT1^+^ stained cells (cortical-neuroendocrine cells) and medullary cells (M), which exhibit a faint positive SYT1 staining. In 6 out the 16 evaluated normal adrenal samples, SYT1^+^ cells formed groups of cells located in the subcapsular region (C-H; ZG = Zona glomerulosa, C = Capsule). All images were acquired by Leica Aperio Versa brightfield scanning microscope (Leica, Germany). Scale bar = 100 µm. I) Evaluation of the SYT staining by automated image analysis (Aperio ImageScope software) showing that SYT1^+^ cells exhibit significantly stronger staining compared to the overall cortex and medulla. annotations “*“and “****” have been used to denote p values less than 0.05 and 0.0001 in figure.


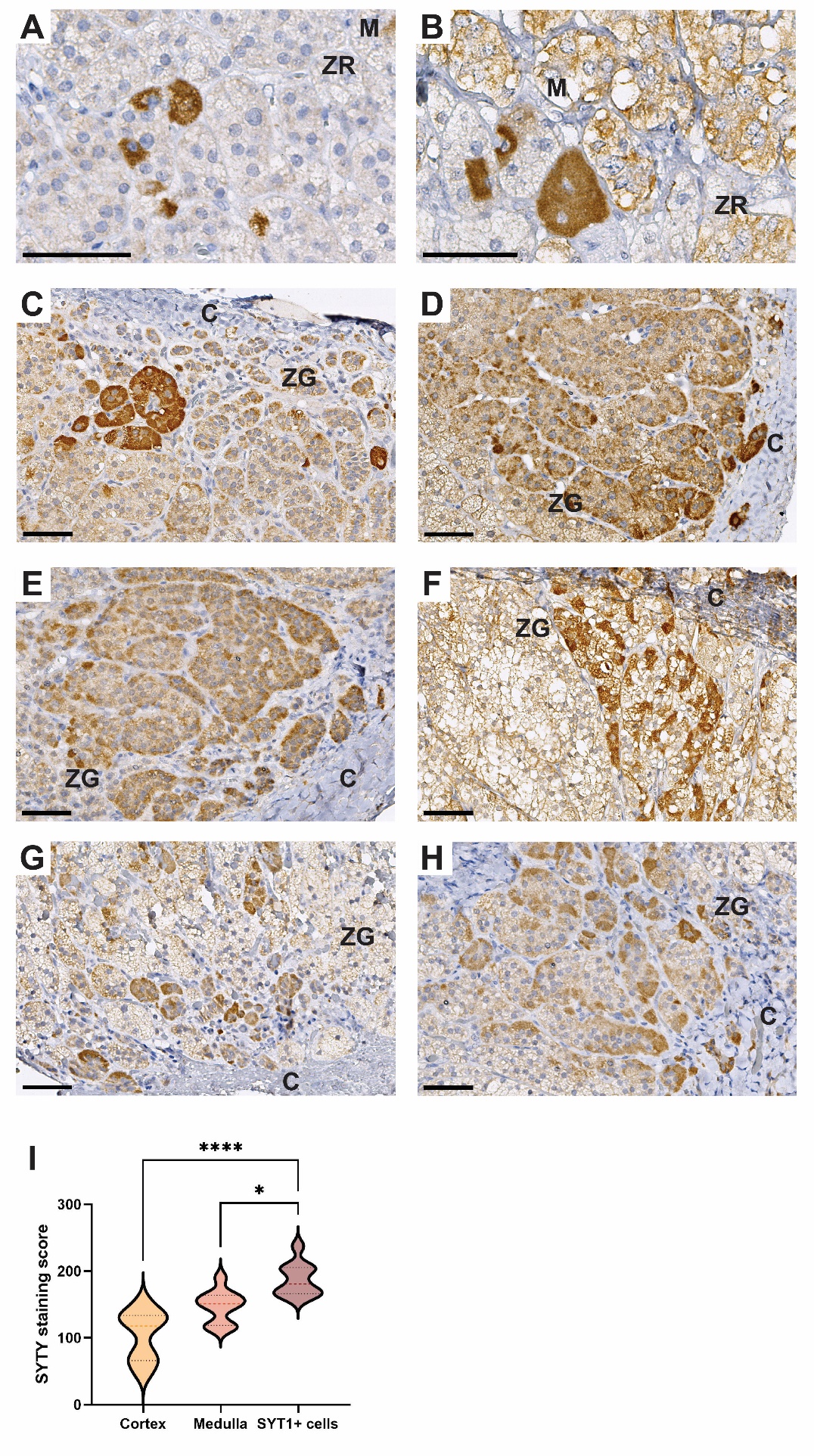


**Supplemental Figure 7. Immunofluorescence for co-staining of CYP17A1 and SYT1.**

Co-staining of CYP17A1 (green) and SYT1 (red) was performed to better characterize the SYT1^+^ cells. Very rare sporadic SYT1^+^-CYP17A1^+^ (yellow) were found within the adrenal cortex of few normal adrenal samples (Figure A-B). A -Region 2) The two antibodies can distinguish very well the adrenal cortex (positive for CYP17A1) from the medulla (positive for SYT1). Images were acquired by Leica Aperio Versa brightfield scanning microscope (Leica, Germany). Scale bar = 100 µm. Abbreviation: ZR = Zona reticularis


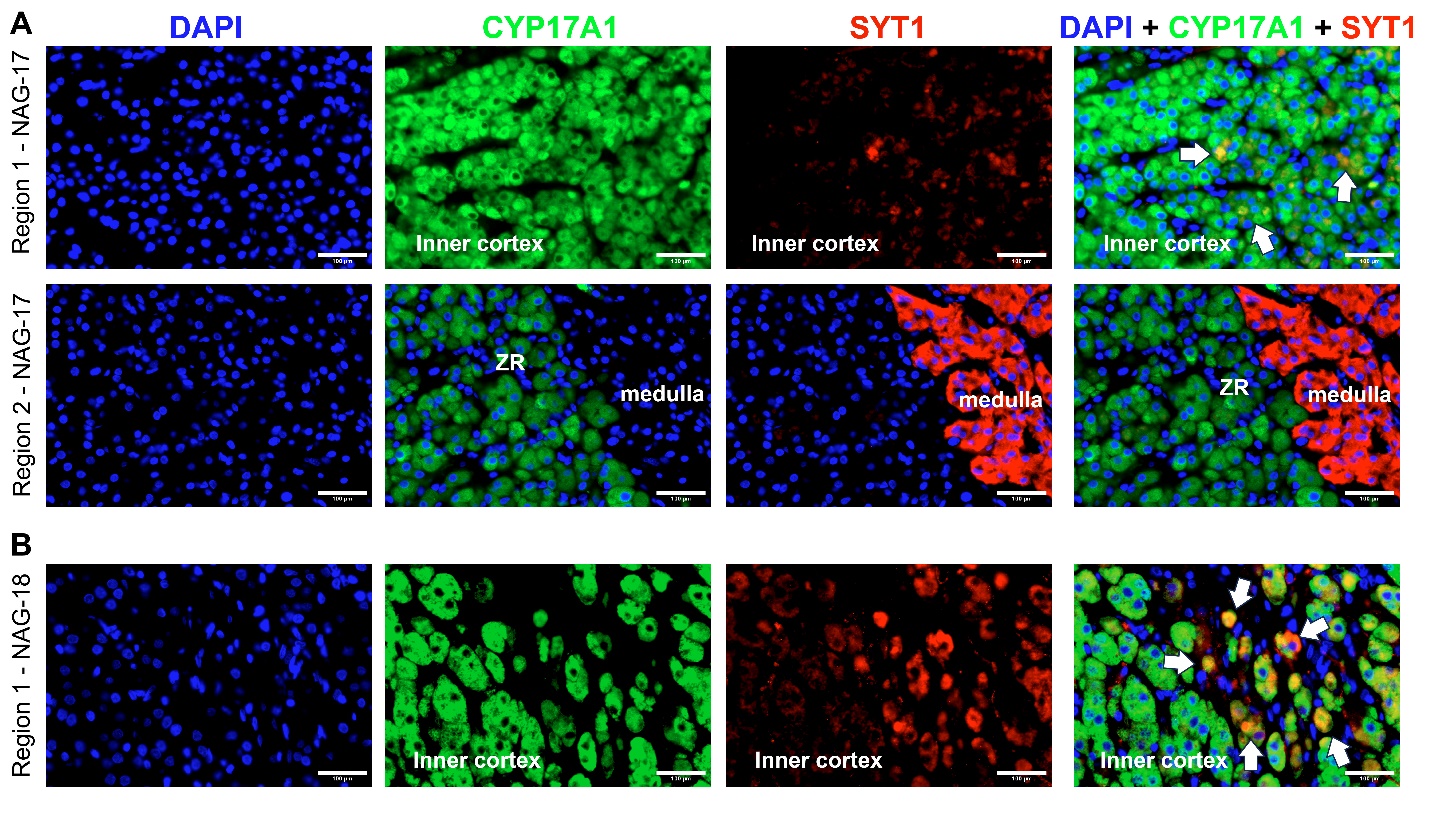


**Supplemental Figure 8. Spatial gene expression plots**

Spatial gene expression plots in both replicates (**A**, **B**), representing expressed genes specific for different clusters: zona glomerulosa (ZG), zona fasciculata (ZF), zona reticularis (ZR), vascular-endothelial cells (VEC), myeloid cells, lymphoid cells, fibroblasts and connective tissue.

**
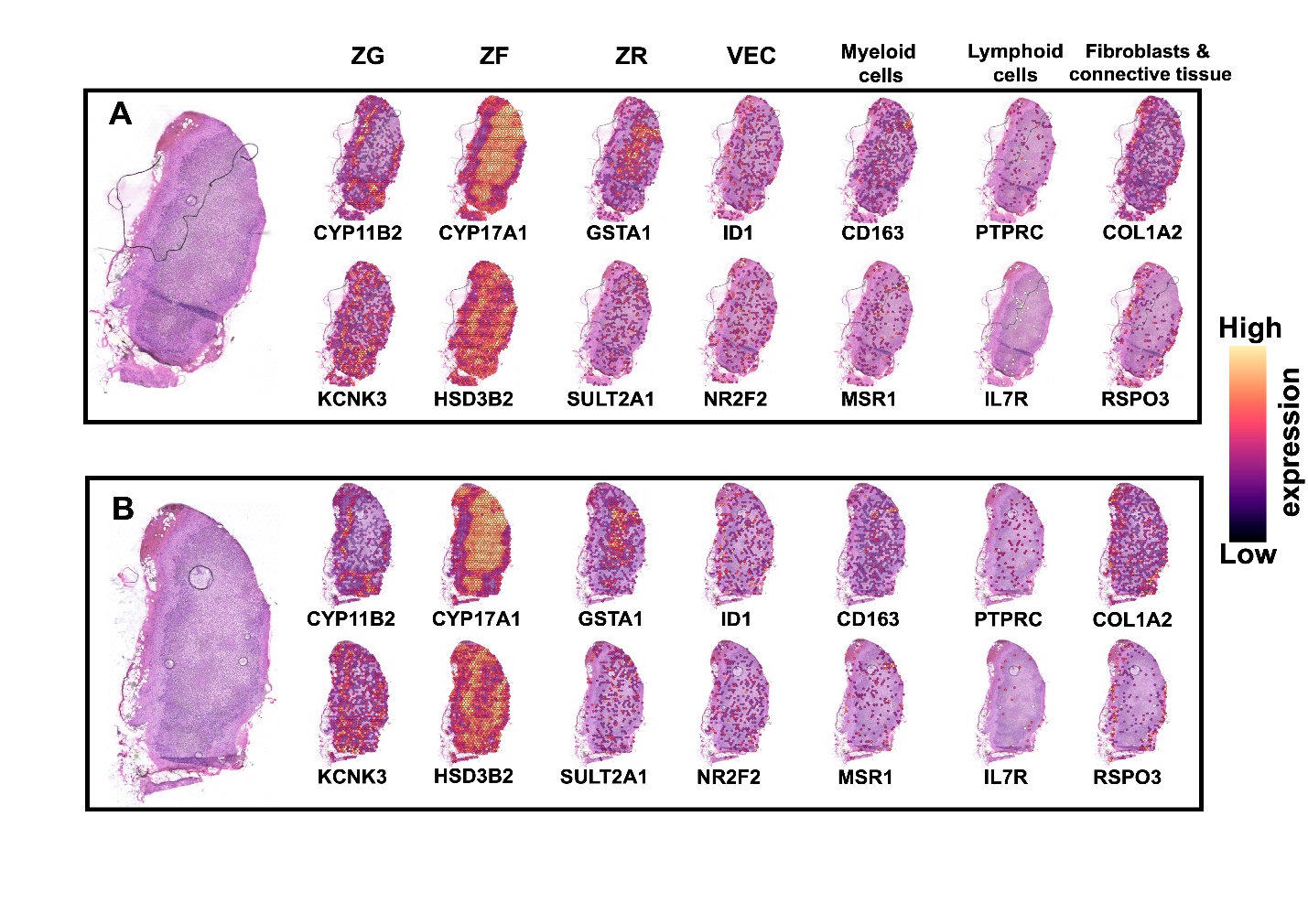
**

**Supplemental Figure 9. Expression of important genes and key pathways genes involved in the adrenal development across pseudotime**

Smoothed lines are generated based on the scatter profile of each gene (95% confidence interval displayed in grey around the line). Vertical lines represent the transition zones from 0-Capsule (red) to 1-ZG (zona glomerulosa, green) and 1-ZG to 2-ZF-ZR (zona fasciculata-reticularis, ochre). Miscellaneous genes are highlighted within the grey box, Wnt/ß-catenin genes are within the blue box, Sonic Hedgehog (SHH) related genes are within the green box, and fibroblast growth factor (FGF) related genes are within the red box. Gene sets for the Wnt, SHH and FGF pathways were acquired from MSigDB and subsequently employed for calculating module scores. The *AddModuleScore* function within Seurat was utilized for this purpose, as described in the Methods section.


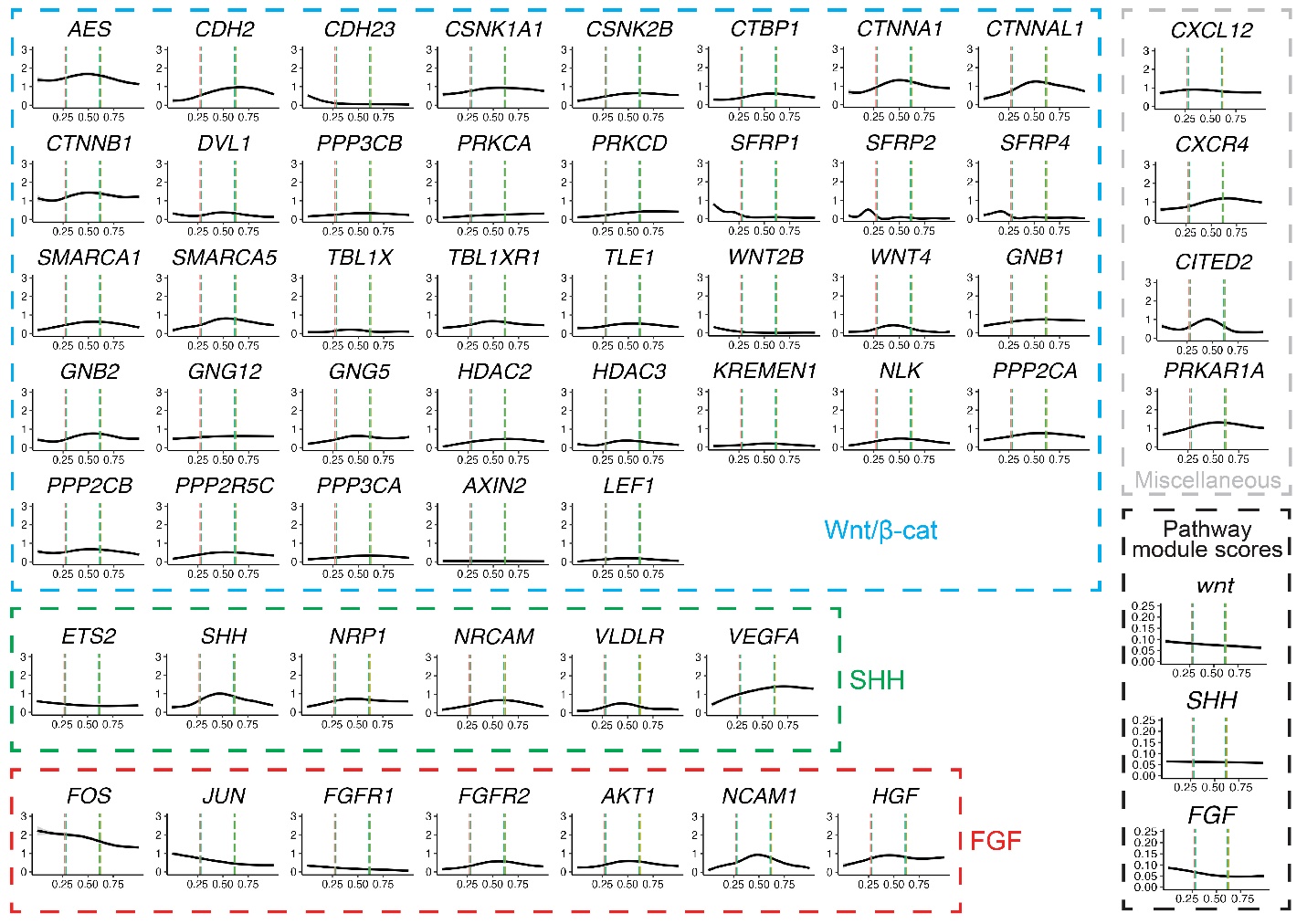


**Supplemental Figure 10. *DLK1* expression across pseudotime**

**A.** Scatter plot representing variation of log normalized expression of DLK1 throughout pseudotime. The highest expression was found in the zona glomerulosa (ZG) to zona fasciculata (ZF) – zona reticularis (ZR) transition (highlighted by a yellow box). Vertical lines represent the transition zones from 0-Capsule (red) to 1-ZG (green) and 1-ZG to 2-ZF-ZR (ochre). **B**. Integrated UMAPs (Uniform Manifold Approximation and Projection) from both Visium sections (10X Genomics) showing the pseudotime trajectory (indicated by black line) and DLK1 expression. **C.** Visium sections showing the DLK1 expression and the previously acquired label transferring clustering in **D.**


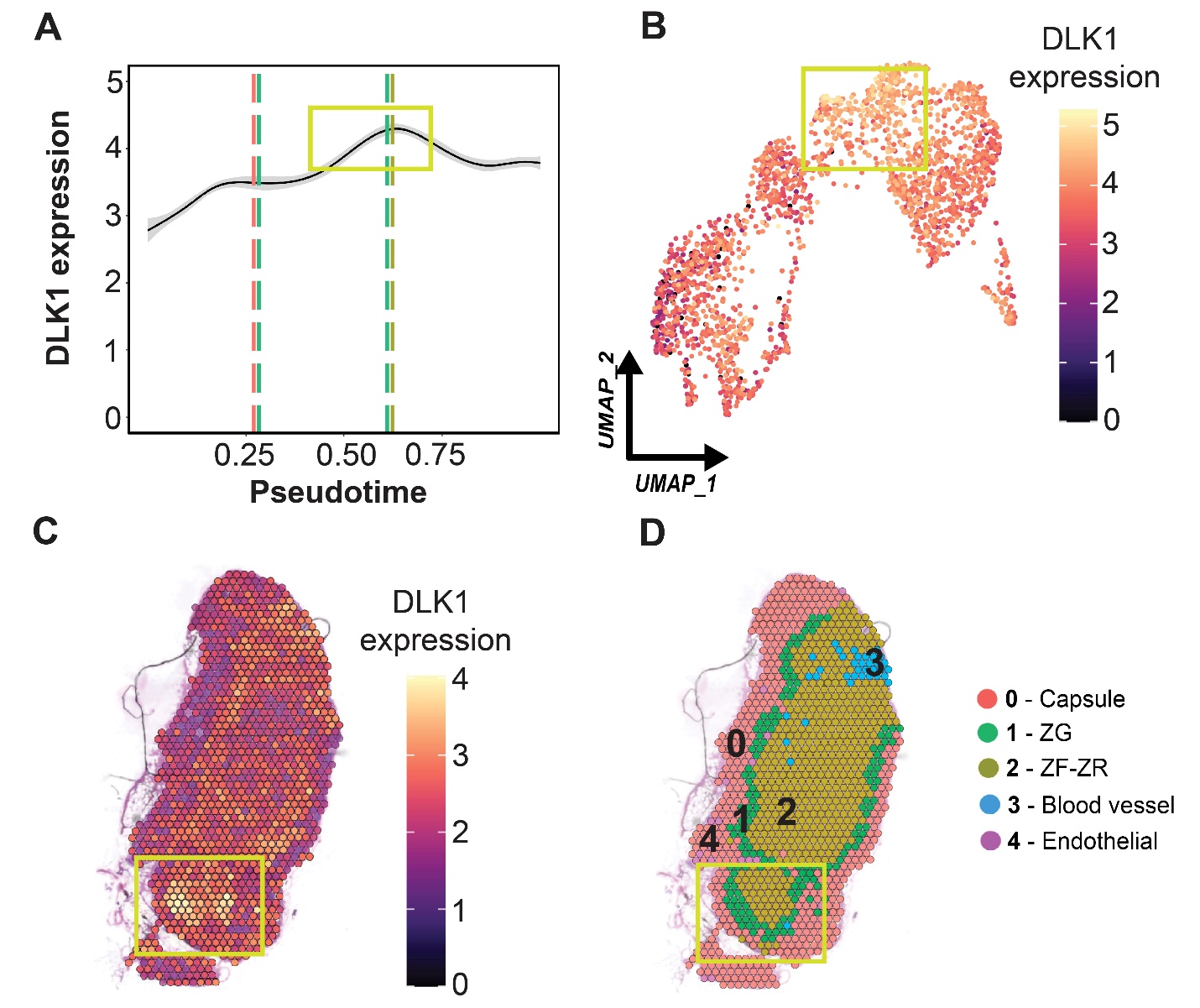


**Supplemental Figure 11. Transcriptomic profile of the newly identified adrenocortical adenoma clusters**

**A.** Heatmap representing top identified features as well as key steroidogenesis markers for the 7 newly identified adenoma clusters. Colour scale represents Z-score computed from log normalized expression matrix. **B.** Top 5 significant pathways for each cluster combining results from 3 separate databases (KEGG, Reactome and GO-All (GO-MF, GO-BP, GO-CC)).


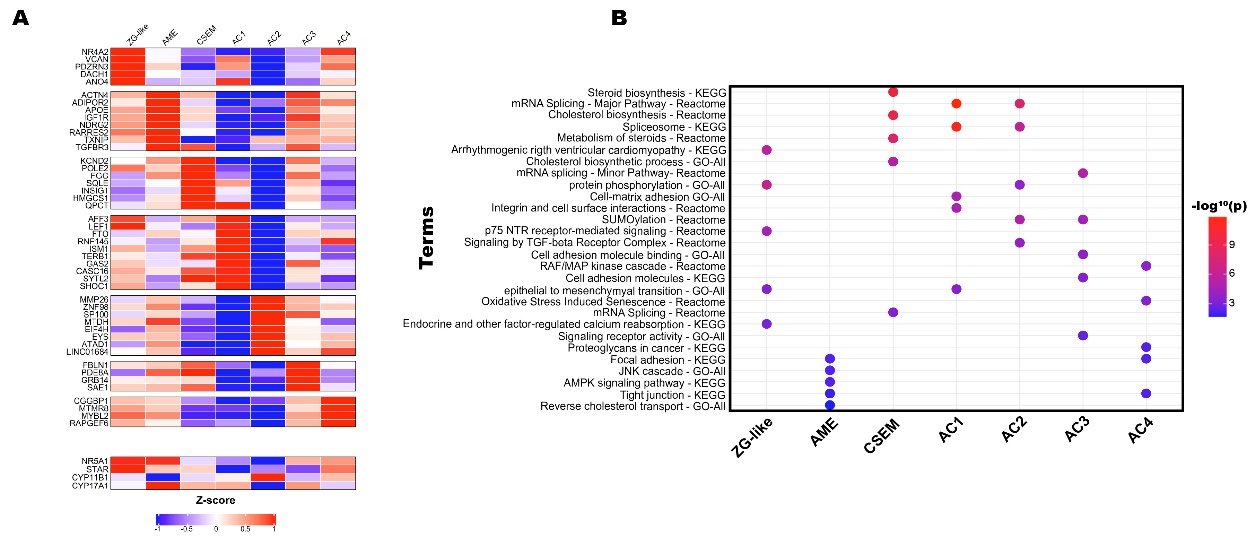


**Supplemental Figure 12. Gene set enrichment analysis of 7 newly identified clusters in adrenocortical adenomas**

Gene set enrichment analysis was performed using pathfindR, using three databases (KEGG, Reactome, GO-All). Curated lists of terms are represented for each of the 7 clusters that were identified in adrenocortical adenomas: similar terms (including Splicing, Extracellular matrix, Cell proliferation and tissue development, Ubiquitin related, Senescence, Cell signalling, receptor tyrosine kinases, Cell Cycle, Cholesterol trafficking, ABC transporters, Cholesterol metabolism, Steroidogenesis, Calcium signalling, Aldosterone synthesis, Other) were placed in groups based on their shared gene lists.


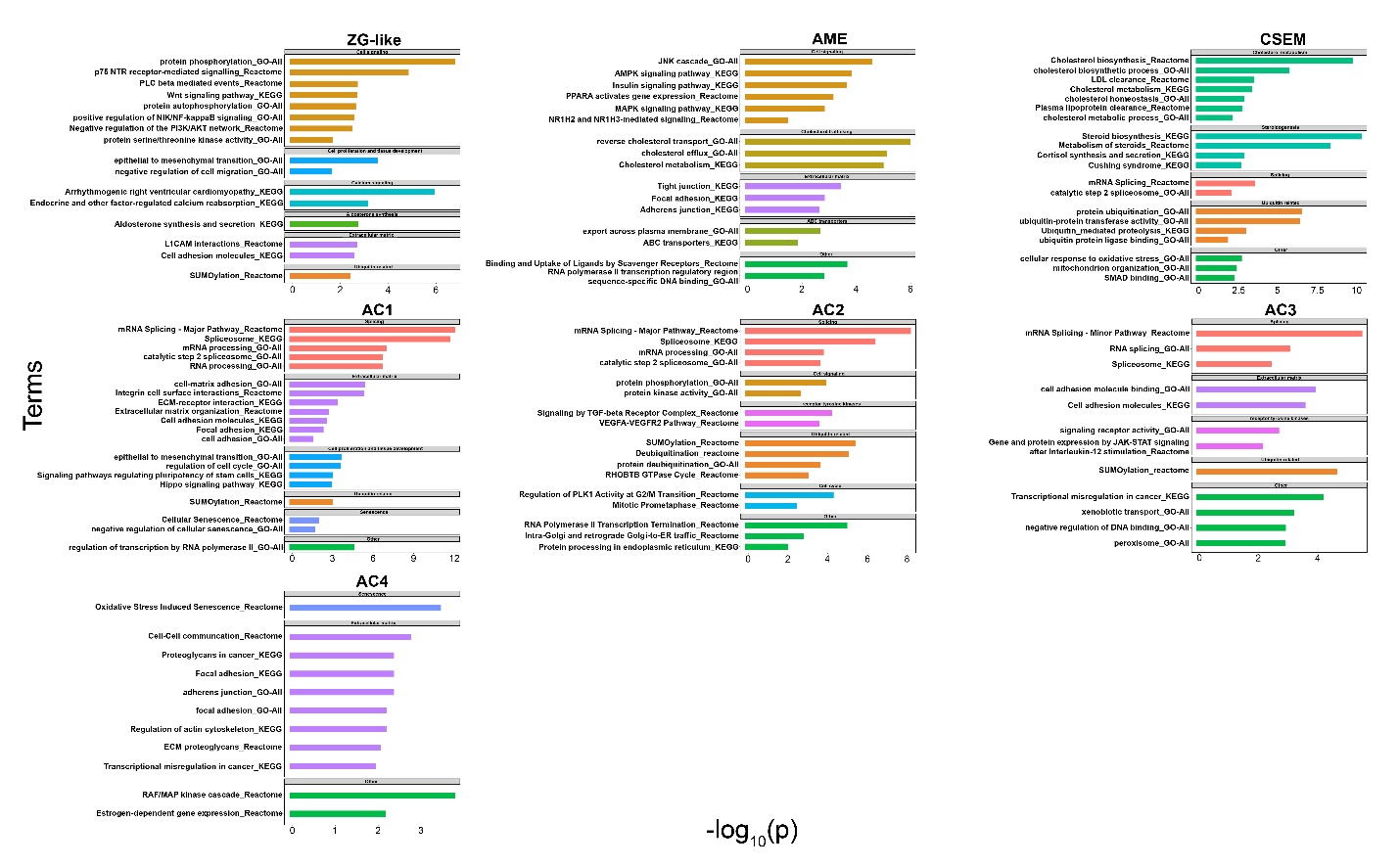


**Supplemental Figure 13. Comparison of the immune transcriptome across normal adrenal glands and subtype adrenocortical adenomas**

Heatmap representing differentially expressed genes (DEGs) across normal adrenal gland (NAG) and subtypes of adrenocortical adenomas based on hormone secretion in endocrine-inactive adenomas (EIA) and cortisol-producing adenoma (CPA). **A.** Lymphoid cells and **B.** myeloid cells.

Hierarchical clustering was performed on the genes (rows) and the average expression values were scaled accordingly.


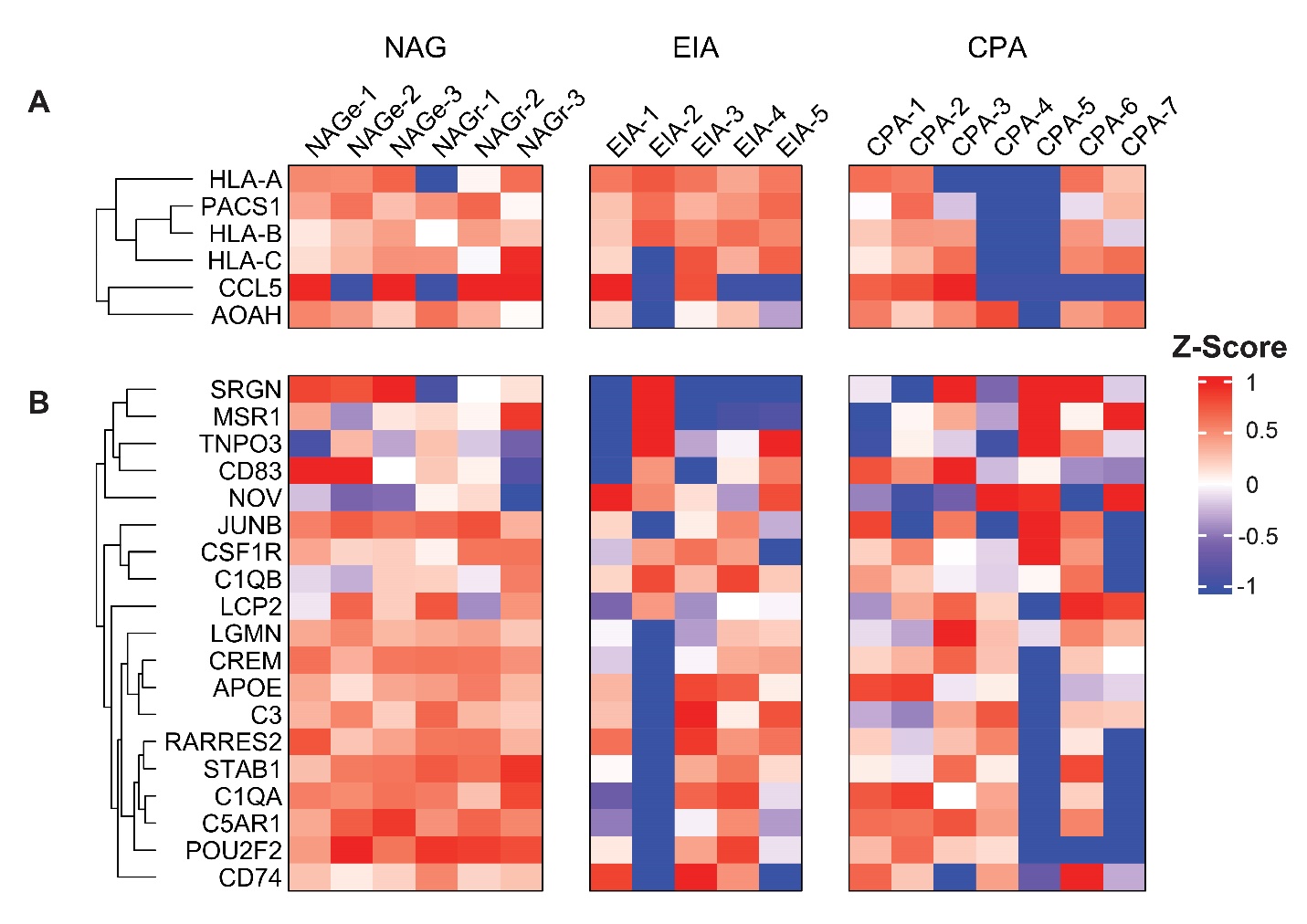


**Supplemental Tables (**tables supplied as a separate excel file with 6 sheets**)**

**Supplemental Table 1. Top 100 differentially up-regulated genes among the clusters of adult human normal adrenal glands**

List of top hundred differentially expressed genes (DEGs) among the different clusters of the normal adrenal glands. Clustering was done at *resolution=0.01* using the *FindClusters* function (as detailed in Methods).

**Supplemental Table 2. Top 100 differentially up-regulated genes among the clusters of adult human adrenocortical adenomas**

List of top hundred differentially expressed genes (DEGs) among the different clusters of adrenocortical adenomas. Clustering was done at *resolution=0.01* using the *FindClusters* function (as detailed in Methods).

**Supplemental Table 3. Details on clinical findings, imaging and histological characteristics of the adrenocortical adenomas**

Abbreviation: AC1-4, adenoma cluster 1-4; CPA, cortisol.-producing adenoma; CSEM, cholesterol-and steroid-enriched metabolism; CT, computed tomography; EIA, endocrine-inactive adenoma; FDG-PET/CT, fluorodeoxyglucose (FDG)-positron emission tomography (PET)/computed tomography (CT); HPF, high-power field; HU, Hounsfield unit; na, not available; RNA-Seq, RNA-sequencing (data from previous study (1)); WES, whole exome sequencing (data from previous study (8))

**Supplemental Table 4. Summary of normal adult adrenal glands evaluated by immunohistochemistry**

Abbreviation: EIA, endocrine-inactive adenoma; F, female; M, male; n.a., not available; NAGe, normal adrenal gland from the tissue surrounding EIA; NAGr, normal adrenal gland from adrenalectomies performed during surgery for RCC; RCC, renal cell carcinoma

**Supplemental Table 5. Primary antibodies used for the immunohistochemistry**

**Supplemental Table 6. PCR primer sequences used for detecting the hot-spot mutation in *CTNNB1, PRKACA and GNAS***

Primers used for the evaluation of known drivers hot-spot mutations in *CTNNB1* (entire exon 3), *PRKACA* (p.Leu206 in exon 7) and *GNAS* (p.Arg201 and p.Gln227) (1, 9) by Sanger sequencing.

Abbreviation: PCR, Polymerase Chain Reaction.

**References to supplemental** **materials**

1. Di Dalmazi G, Altieri B, Scholz C, Sbiera S, Luconi M, Waldman J, et al. RNA Sequencing and Somatic Mutation Status of Adrenocortical Tumors: Novel Pathogenetic Insights. *J Clin Endocrinol Metab.* 2020;105(12).

2. Geiger J, Both S, Kircher S, Neumann M, Rosenwald A, and Jahns R. Hospital-integrated Biobanking as a Service – The Interdisciplinary Bank of Biomaterials and Data Wuerzburg (ibdw). *Open Journal of Bioresources.* 2018;5.

3. Detomas M, Deutschbein T, Tamburello M, Chifu I, Kimpel O, Sbiera S, et al. Erythropoiesis in Cushing syndrome: sex-related and subtype-specific differences. Results from a monocentric study. *Journal of Endocrinological Investigation.* 2023.

4. Nieman LK, Biller BMK, Findling JW, Newell-Price J, Savage MO, Stewart PM, et al. The Diagnosis of Cushing's Syndrome: An Endocrine Society Clinical Practice Guideline. *The Journal of Clinical Endocrinology & Metabolism.* 2008;93(5):1526-40.

5. Hashimshony T, Senderovich N, Avital G, Klochendler A, de Leeuw Y, Anavy L, et al. CEL-Seq2: sensitive highly-multiplexed single-cell RNA-Seq. *Genome Biology.* 2016;17(1):77.

6. Klein AM, Mazutis L, Akartuna I, Tallapragada N, Veres A, Li V, et al. Droplet barcoding for single-cell transcriptomics applied to embryonic stem cells. *Cell.* 2015;161(5):1187-201.

7. Krishnaswami SR, Grindberg RV, Novotny M, Venepally P, Lacar B, Bhutani K, et al. Using single nuclei for RNA-seq to capture the transcriptome of postmortem neurons. *Nat Protoc.* 2016;11(3):499-524.

8. Ronchi CL, Di Dalmazi G, Faillot S, Sbiera S, Assié G, Weigand I, et al. Genetic Landscape of Sporadic Unilateral Adrenocortical Adenomas Without PRKACA p.Leu206Arg Mutation. *J Clin Endocrinol Metab.* 2016;101(9):3526-38.

9. Ronchi CL, Di Dalmazi G, Faillot S, Sbiera S, Assie G, Weigand I, et al. Genetic Landscape of Sporadic Unilateral Adrenocortical Adenomas Without PRKACA p.Leu206Arg Mutation. *J Clin Endocrinol Metab.* 2016;101(9):3526-38.
